# Inhibition of AKT enhances chemotherapy efficacy and synergistically interacts with targeting of the Inhibitor of apoptosis proteins in oesophageal adenocarcinoma

**DOI:** 10.1038/s41598-024-83912-4

**Published:** 2024-12-30

**Authors:** Leanne Stevenson, Lauren Cairns, Xiaodun Li, Sriganesh Jammula, Harriet Taylor, Rosalie Douglas, Niamh McCabe, Gerald Gavory, Xavier Jacq, Rebecca C. Fitzgerald, Richard D. Kennedy, Timothy Harrison, Richard C. Turkington

**Affiliations:** 1 Patrick G Johnston Centre for Cancer Research, Queen’s University Belfast, Belfast, Northern Ireland; 2 MRC Cancer Unit, Hutchison/MRC Research Centre, University of Cambridge, Cambridge, UK; 3 Almac Discovery Ltd, Craigavon, Northern Ireland; 4 Almac Diagnostics Ltd, Craigavon, Northern Ireland

## Abstract

The incidence of oesophageal adenocarcinoma (OAC) has risen six-fold in western countries over the last forty years but survival rates have only marginally improved. Hyperactivation of the PI3K-AKT-mTOR pathway is a common occurrence in OAC, driving cell survival, proliferation and resistance to chemotherapeutic agents. Inhibition of AKT has been explored as a treatment strategy with limited success and current inhibitors have failed to progress through clinical trials. Our study, describes a novel allosteric AKT inhibitor, ALM301, and demonstrates an enhancement of the efficacy of conventional chemotherapy when combined with ALM301 in OAC. Reduced sensitivity to ALM301 is associated with high expression of the Inhibitor of Apoptosis (IAP) family of proteins, particularly XIAP. Combined AKT and IAP inhibition synergistically enhanced OAC cell death and successfully re-sensitized ALM301 and chemotherapy resistant cell lines. A high degree of synergism was also observed in patient-derived OAC organoids indicating the potential clinical relevance of the combination. This study demonstrates the role for dual AKT/IAP inhibition in OAC and provides a strong rationale for the further investigation of this highly efficacious combination strategy.

## Introduction

Incidence rates of OAC have progressively increased by six-fold since the 1970s, with the United Kingdom reported to have one of the highest global incidence rates together with one of the poorest survival rates^1^. Standard therapy for resectable OAC includes either neo-adjuvant chemotherapy or chemoradiotherapy followed by surgical resection in patients with localized disease. Despite optimal therapy these patients have a 5-year survival rate of only 36–47%^2,3^. However, OAC is an aggressive disease with early loco-regional spread and most patients present with advanced, inoperable, or metastatic disease and may be eligible for palliative chemotherapy with 5-Fluoruracil (5-FU) and Cisplatin (cis-diaminodichloroplatinum -CDDP) or oxaliplatin-based regimens. The 5-year survival for these patients is poor at 13%^4,5^. Consequently, new treatment approaches are vital to improve survival rates in OAC.

The serine/threonine kinase AKT, is encoded by three closely related genes, AKT1, AKT2, and AKT3 and is pivotal to phosphoinositide 3-kinase (PI3K)-AKT-mTOR pathway signaling. Hyper-activation of this pathway is common across many cancer types and has been associated with resistance to standard chemotherapies, targeted agents and radiotherapy^6–8^. This can arise through multiple mechanisms including PTEN loss or mutation, PI3K or AKT mutation or, more commonly, upstream receptor tyrosine kinase (RTK) amplifications or activation^9^. Whole genome sequencing studies have identified OAC as a disease dominated by copy number alterations with 43% of tumors harboring gains/amplifications of upstream RTKs^10^. Moreover, RTK pathways are dysregulated by driver mutations and/or copy number variations (CNVs) in 60% of OAC tumors^11^. PI3K/AKT is also identified as the most commonly mutated oncogenic pathway; therefore AKT is an attractive therapeutic target in this disease^12^.

Different chemical classes of small-molecule AKT inhibitors with varying potency and isoform specificity have been developed^13^. Allosteric inhibitors of the AKT Pleckstrin homology (PH)-domain prevent localization of AKT to the plasma membrane, thereby blocking AKT phosphorylation and activation, whereas ATP-competitive inhibitors of AKT exert their mechanism of action through targeting of the ATP-binding pocket of AKT. However, AKT inhibitors have, to date, failed to progress through clinical trials due to lack of clinical activity as a monotherapy, dosing and scheduling difficulties or overlapping toxicity with certain drug combinations^14^.

ALM301 is a novel allosteric small molecule inhibitor of AKT which potently and selectively inhibits AKT1/2 and has demonstrated efficacy in tumour growth and preclinical xenograft models^15^. We sought to assess the role of AKT inhibition in sensitization of OAC cells to chemotherapy and to identify novel combination strategies to overcome intrinsic resistance using the allosteric AKT inhibitor, ALM301. We show that resistance to AKT inhibition is regulated by expression of the IAP family of proteins and that targeting IAPs with the SMAC mimetic BV6 synergizes with ALM301. Dual AKT/IAP inhibition may represent a novel combination treatment strategy to overcome intrinsic and acquired resistance to AKT inhibition and is also effective in acquired chemotherapy resistance in OAC.

## Results

### AKT inhibition enhances the anti-tumor activity of chemotherapy in OAC cell lines

**Fig. 1:**
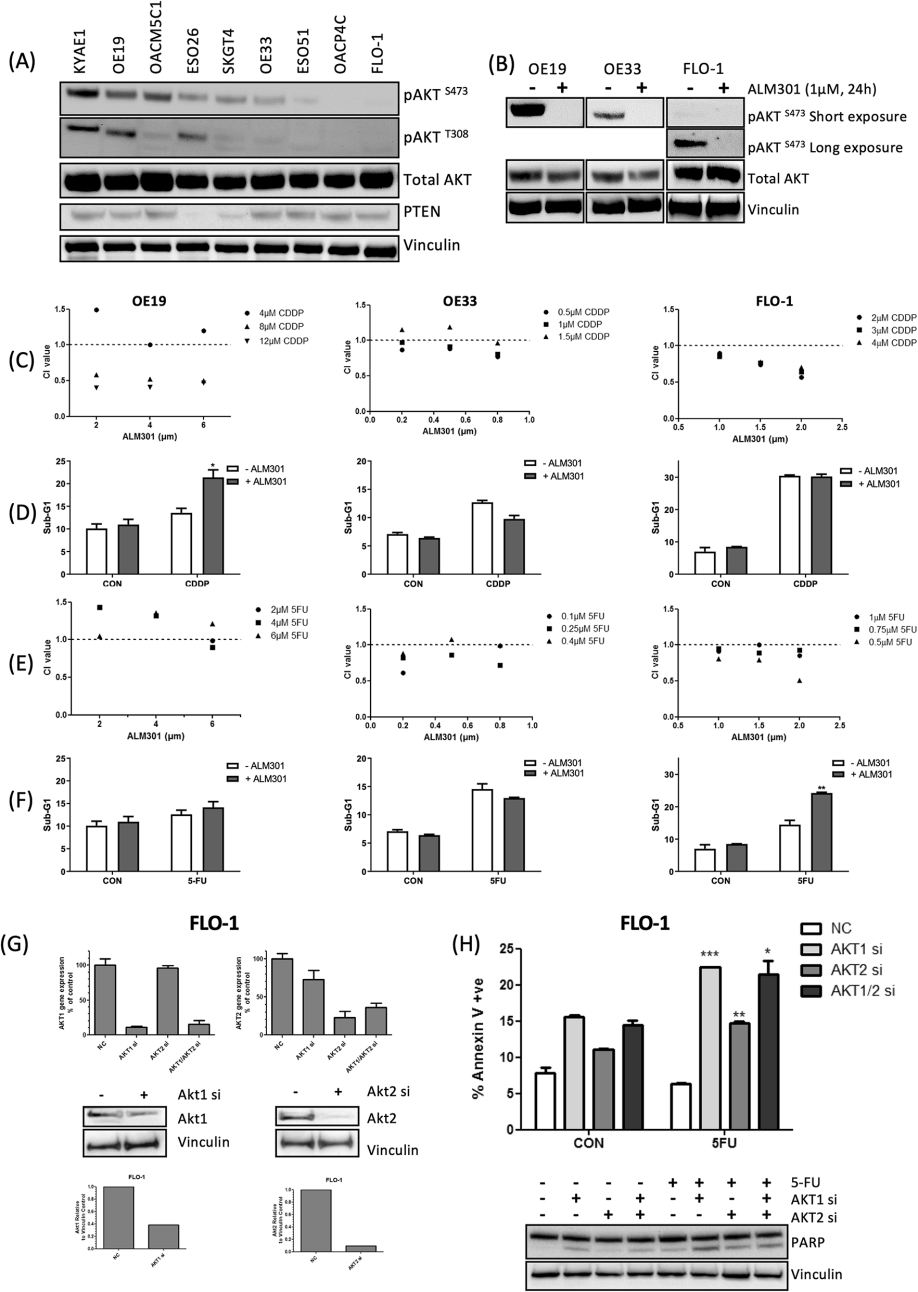
AKT inhibition enhances anti-tumour activity of chemotherapy. (**A**) Western blot analysis of constitutive levels of AKT protein expression/phosphorylation and PTEN protein in EAC cell line panel. (**B**) phospho-AKT-S473 to confirm of AKT inhibition in OAC cell lines following 24 h treatment with 1 µM ALM301. Vinculin was used as a loading control. (**C**) Combination index (CI) values from MTT viability assays following 72 h treatment with ALM301/CDDP combinations using drug doses of ~ IC_30(72 h)_ or less. A CI value < 1, = 1, and > 1 indicates synergism, additivity and antagonism respectively. (**D**) Flow cytometric analysis of the sub-G1 OAC cell population at 72 h post-treatment with ~ IC_30(72 h)_ doses of ALM301 or CDDP alone or in combination. (**E**) Combination index (CI) values from MTT viability assays following 72 h treatment with ALM301/5-FU combinations using drug doses of ~ IC_30(72 h)_ or less. (F) Flow cytometric analysis of the sub-G1 OAC cell population at 72 h post-treatment with ~ IC_30(72 h)_ doses of ALM301 or CDDP alone or in combination. A 2-way ANOVA was used to test for statistical significance of the ALM301/BV6 combination where NS is not significant, *** = p < 0.001, ** = p < 0.01 and * = P < 0.05. Data is representative of the mean of triplicate experiments ± standard error of the mean (SEM). (**G**) Q-PCR and Western blot analysis of AKT1 and AKT2 to confirm AKT knockdown at the gene and protein level respectively. Densitometry was applied to quantify levels of AKT. (**H**) Flow cytometric analysis of the Annexin V-positive FLO-1 cell population following AKT1/2 silencing alone or in combination with 72 h ~ IC_30(72 h)_ 5-FU treatment. Western blot analysis of cleaved PARP following AKT1/2 silencing alone or in combination with 72 h ~ IC_30(72 h)_ 5-FU treatment. Vinculin was used a loading control. Statistical significance of 5-FU-treated compared untreated controls was assessed by a paired t-test where NS = not significant, *** = p < 0.001, ** = p < 0.01 and * = p < 0.05. Data is representative of the mean ± SEM.

Given the poor survival rates of OAC patients receiving chemotherapy, we first asked if AKT inhibition could improve OAC cell line sensitivity to the standard chemotherapies, 5-FU and CDDP. Western blot analysis was used to characterize a panel of 9 OAC cell lines for basal AKT protein expression and phosphorylation levels of 2 key AKT residues, T308 of the catalytic core and S473 of the C-terminal hydrophobic motif (Fig. 1A. Three cell lines were selected for further in-depth analysis based on high (OE19), medium (OE33) and low (FLO-1) phospho-AKT (pAKT) levels and western blot analysis of pAKT-S473 confirmed AKT inhibition following 24 h treatment with 1 µM ALM301 (Fig. 1B).

An MTT dose–response assay was used to measure cell viability at 72 h post-treatment and calculate ~ IC_30(72 h)_ doses of ALM301, 5-FU and CDDP (Supplementary Table S1). ALM301 and chemotherapy were then combined at doses ≤ IC_30(72 h)_ and an MTT assay was used to assess viability at 72 h post-treatment. Combination index (CI) values showed synergy (CI < 1) of ALM301/CDDP in all 3 cell lines (Fig. 1C). Sub-G1 analysis, an indicator of apoptosis, showed ALM301 significantly interacted with CDDP in the OE19 cells (P < 0.05 Fig. 1D) but not in the OE33 or FLO-1 cells.

CI values showed ALM301/5-FU synergy in the FLO-1 and OE33 cell lines (CI < 1) but not in the OE19 cells (CI > 1) (Fig. 1E). Sub-G1 analysis, showed significant ALM301/5-FU interaction in the FLO-1 cells but not in the OE19 or OE33 cells (P < 0.01 Fig. 1F). The Sub-G1 results suggest that ALM301/CDDP synergy in OE19 and ALM301/5-FU synergy in the FLO-1 cells is attributable to enhanced cell death. This was further validated by an increase in Annexin V positive cells (Supplementary Fig. S1A), together with increased cleaved PARP, an indicator of apoptosis (Supplementary Fig. S1B). Of note, 5-FU-induced pAKT-S473 by was observed in the FLO-1 cells, which have constitutively low pAKT-S473 levels. This would suggest that AKT activation mediates resistance to 5-FU treatment in this in vitro setting. In the OE19 cells, which have constitutively high pAkt-S473, no further increase in phosphorylation was observed following CDDP treatment. The Sub-G1 results also suggest that ALM301/CDDP synergy observed in OE33 and FLO-1 cells and ALM301/5-FU synergy observed in the OE33 cells is not attributable to cell death and therefore may be a result of cytostasis. This was further validated by significantly decreased proliferation rates (Supplementary Fig. S1C).

To assess if lack of ALM301/5-FU synergy in OE19 cells was simply due to decreased drug influx, increased efflux or drug inactivation, all of which are known mechanisms of drug-resistance which prevent chemotherapy from inducing DNA damage; phospho-γH2AX-S139, a hallmark of DNA damage, was examined by western blot (Supplementary Fig. S1D). This confirmed 5-FU-induced DNA damage in the OE19 cells suggesting that the lack of ALM301 synergy was not attributable to drug influx/efflux or inactivation and is therefore mediated by another mechanism. ALM301/chemotherapy synergy was also demonstrated by 2D-clonogenic assay in the OE33 and FLO-1 cells (Supplementary Fig. S1).

Since allosteric AKT inhibitors do not directly target the conserved kinase domain, they are considered to have higher target specificity and therefore fewer off-target effects than ATP-competitive inhibitors^16^. ALM301-induced chemo-sensitization was confirmed as an on-target effect of AKT inhibition by targeting AKT1 and AKT2 with siRNA in combination with 5-FU in the FLO-1 cells. AKT1 and AKT2 knockdown was confirmed by Q-PCR and Western blot analysis (Fig. 1G). Knock down of AKT1 or AKT2 alone or in combination, significantly interacted with 5-FU to enhance cytotoxicity (P < 0.001, 0.01 and 0.05 respectively) and this was reflected by increased cleaved PARP (Fig. 1H).

Taken together, these results show that ALM301 has the potential to synergize with chemotherapy in OAC cell lines through enhanced cytotoxicity or abrogated cell proliferation. Importantly, the lack of ALM301/5-FU synergy in the OE19 cells highlights that there is a dependence on context. We therefore sought to investigate potential mechanisms for these differential responses.

### Chemotherapy modulates IAP protein expression in OAC cell lines

**Fig. 2:**
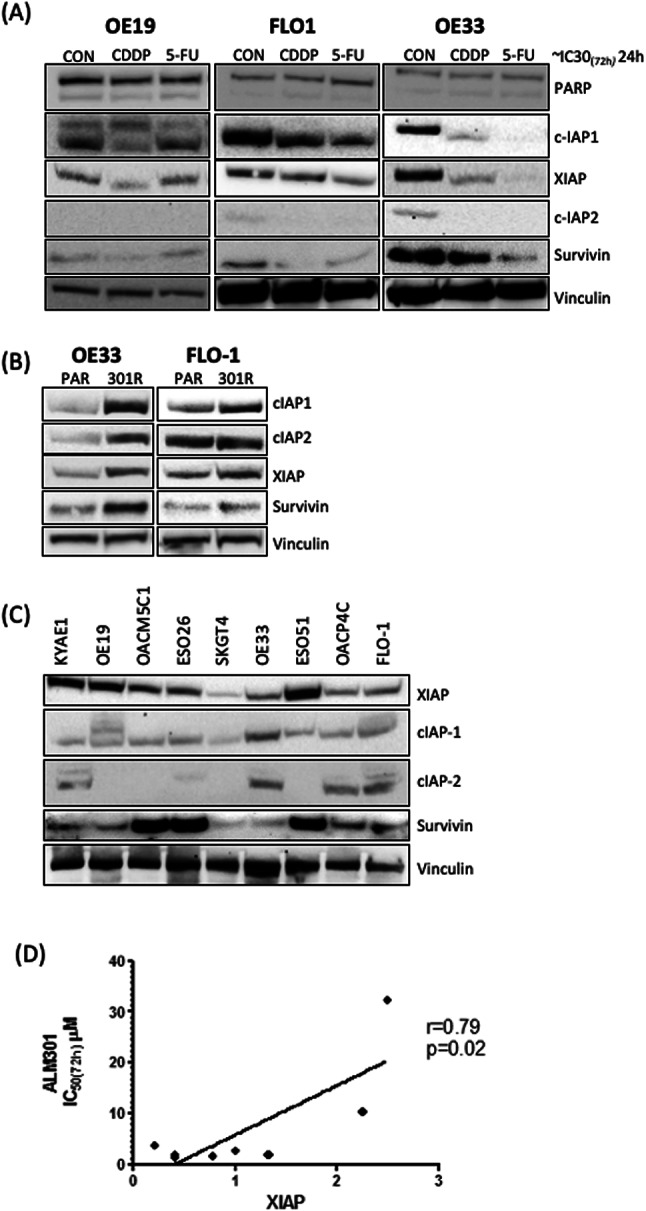
Characterisation of IAP proteins in OAC cells. (**A**) Western blot analysis of cleaved PARP, cIAP1, cIAP2, XIAP and Survivin following 24 h treatment with ~ IC_30(72 h)_ doses of chemotherapy in OE33, FLO1 and OE19 cell lines. Western blot analysis of basal IAP protein levels in (**B**) paired sensitive and resistant OAC cell lines and (**C**) an OAC cell line panel. Vinculin was used as a loading control (**D**) Pearson correlation analysis of wild-type XIAP protein levels and ALM301 sensitivity, ~ IC50_(72 h)_ in the OAC cell line panel (r = 0.79, p < 0.05). XIAP mutant KYAE1 cell line was omitted from the analysis due to predicted functionality effect.

A reverse phase protein analysis study in high grade serous ovarian cancer (HGSOC) identified anti-apoptotic proteins as a mechanism of resistance to inhibitors of the PI3K/Akt/mTOR pathway^17^. IAP proteins are a family of 8 anti-apoptotic proteins that are frequently dysregulated in cancer and contribute to chemo-resistance^18^. Focusing on the most characterized IAP family members, cIAP1, cIAP2, XIAP and Survivin, we asked if differential chemo-regulation of IAPs in OAC cell lines could mediate the context-dependent ALM301-sensitization observed in the OAC cell lines. Chemotherapy regulation of IAP protein expression was assessed by western blot analysis at 24 h post-treatment with ~ IC_30(72 h)_ chemotherapy doses (Fig. 2A). Importantly, PARP cleavage was not enhanced by chemotherapy at this time-point confirming that cytotoxicity was not a cause of protein dysegulation. Interestingly, 5-FU in OE19 cells, the setting that lacked synergy with ALM301, had no effect on IAP expression. Whereas, IAP proteins were acutely down-regulated by the chemotherapy settings that synergized with ALM301. These results were confirmed by gene expression analysis of a cohort of pre-treatment endoscopic OAC biopsies with matched post-treatment surgical resection specimens. All patients were treated with oxaliplatin and 5FU-based neoadjuvant therapy with a significant reduction in cIAP2 (p = 0.011) and Survivin (p = 0.034) expression observed following treatment, in keeping with our observations in OAC cell lines (Supplementary Figure S1).

### IAP proteins are elevated in ALM301-resistant cells

Given that chemotherapy-induced down-regulation of IAPs was associated with sensitization to AKT inhibition, we hypothesized that IAP proteins would be upregulated in ALM301 resistance. ALM301-R-OE33 and -FLO-1 sub-lines were confirmed as more resistant than the parental cell line by 8.6 and 4.1 fold respectively (Table 1). Western blot analysis of basal IAP protein expression revealed that cIAP1, XIAP, and Survivin were indeed upregulated in ALM301-R-OE33 and -FLO-1 cell lines compared to the parental counterparts (Fig. 2B). Whereas cIAP2 was upregulated in the ALM301-R-OE33 but not the ALM301-R-FLO-1 cells.

Next, a comprehensive panel of 9 OAC cell lines were examined for ALM301 sensitivity by Cell Titre Glo® viability assay and ~ IC_50(72 h)_ doses were calculated (Supplementary Table S2). Western blot and densitometry analysis were used to assess basal IAP protein expression (Fig. 2C, Supplementary Table S3). Pearson analysis was used to determine if intrinsic ALM301 resistance correlated with high basal IAP protein levels (Supplementary Table S3). No significant correlation was observed for basal cIAP1, cIAP2, XIAP or Survivin levels (p < 0.05). However, the KYAE-1 cells harbor a XIAP E350delE mutation and this is predicted to have a functional impact^19^. When the KYAE-1 cell lines were omitted from the Pearson analysis, a significant correlation between ALM301 intrinsic resistance and high wild-type XIAP expression was observed in the OAC cell line panel (r = 0.79, p < 0.05) (Fig. 2D). This may suggest that constitutively high XIAP protein levels confer intrinsic resistance to ALM301.

High pAKT-S473 levels are reported to confer AKT inhibitor sensitivity^20^. We therefore examined whether AKT expression or phosphorylation could also play a role in ALM301 sensitivity in the OAC cell line panel. Pearson correlation analysis was applied as above (Supplementary Table S3). Surprisingly, ALM301 sensitivity showed no correlation with total-AKT expression (Pearson *r* = -0.12), pAKT-T308 (Pearson *r* = -0.09), pAKT-S473 (Pearson *r* = -0.21), or combined pAKT-T308/S473 (Pearson *r* = -0.15). The activity of the AKT pathway is counteracted by the PIP3 phosphatase, PTEN. However, all of the OAC cell lines are PTEN wild-type and PTEN expression did not correlate with sensitivity to ALM301. This data suggests that neither PTEN expression nor AKT expression or activity confer sensitivity to ALM301 and are unreliable biomarkers in the OAC cell line models. These findings suggest that IAP proteins mediate both acquired and intrinsic resistance to AKT inhibition.

### Targeting IAP proteins with BV6 depletes cIAP1, cIAP2 and XIAP and synergizes with ALM301 in OAC cell lines

**Fig. 3:**
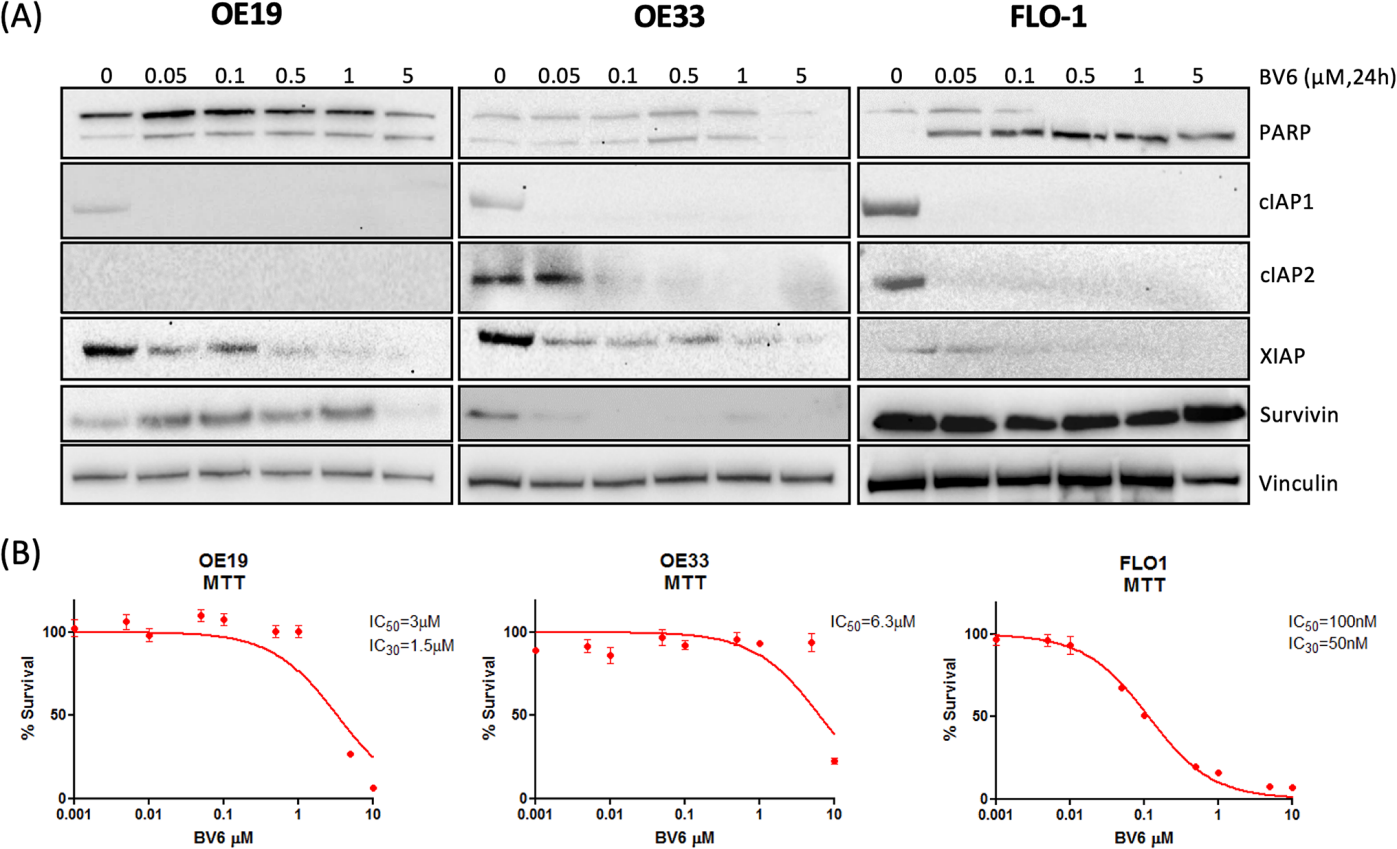
IAP antagonist activity in OAC cells (**A**) Western blot analysis of cleaved PARP, cIAP1, cIAP2, XIAP and Survivin protein levels following 24 h treatment with SMC, BV6. Vinculin was used as a loading control. (**B**) MTT analysis of OAC cell viability following 72 h treatment with BV6.

To confirm if IAP proteins mediate resistance to AKT inhibition, we proposed that targeting IAP proteins with a second mitochondria-derived activator of caspases (SMAC) mimetic compound (SMC), also known as IAP antagonists, would sensitize to ALM301. SMAC is an endogenous antagonist of XIAP, cIAP1, and cIAP2 and IAP antagonists can eliminate IAPs by promoting autoubiquitylation and proteasomal degradation. Western blot analysis following a 24 h dose response with the IAP antagonist, BV6, revealed depletion of cIAP1, cIAP2 and XIAP in the OAC cell lines (Fig. 3A). As noted previously, cIAP2 was not detected in the OE19 cells. A BV6 dose-dependent depletion of Survivin was also observed in the OE33 but not the OE19 or FLO-1 cells. Of note, BV6-induced XIAP depletion was not a consequence of caspase activation since XIAP was also depleted in FLO-1 cells treated with a pan-caspase inhibitor, zVAD-fmk (Supplementary Fig. S4A).

BV6 sensitivity in OE33, FLO-1 and OE19 cells was assessed by MTT assay following a 72 h dose–response (Fig. 3B). FLO-1 cells were the most sensitive to BV6 (~ IC_30(72 h)_ = 50 nM) and this was reflected in dose-dependent elevated cleaved PARP levels (Fig. 3A). Dose-dependent cleaved PARP was not observed in the OE33 or OE19 cells. A 1 µM dose ALM301 was effective at inhibiting pAKT-S473 for 72 h in the OE19 and FLO-1 cells but not the OE33 cells where pAKT-S473 levels increased at 48 h and 72 h compared to 24 h post-treatment (Supplementary Fig. S4B). Interestingly, the ALM301/BV6 combination was more effective at inhibiting pAKT-S473 at the longer time points in the OE33 cells.

**Fig. 4:**
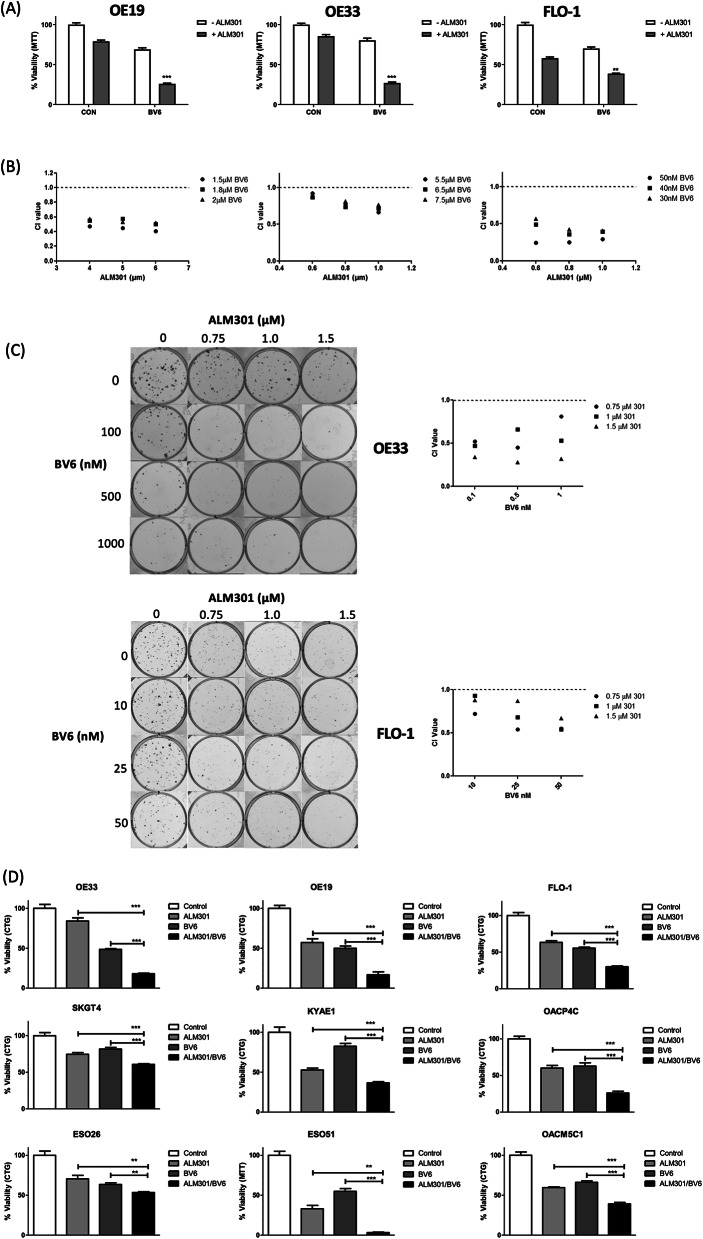
Synergy between AKT inhibitor (ALM301) and IAP antagonist (BV6) in OAC cells. (**A**) MTT analysis of OAC cell viability following 72 h treatment with ~ IC_30(72 h)_ doses of BV6 or ALM301 alone or in combination. Statistical significance of the ALM301/BV6 combination was assessed by a 2-way ANOVA where NS = not significant, *** = p < 0.001, ** = p < 0.01 and * = P < 0.05. Data is representative of the mean of triplicate experiments ± SEM. (**B**) MTT assays were used to assess OAC cell viability at 72 h post-treatment with ALM301/BV6 combinations using drug doses of ~ IC_30(72 h)_ or less. (**C**) Colony formation assays were used to assess OAC cell viability at ~ 10–14 days post-treatment with ALM301/BV6 combinations using drug doses of ~ IC_30(72 h)_ or less. To evaluate the interaction between BV6 and ALM301, the method of Chou and Talalay was used to calculate combination index (CI) values. CI values < 1, = 1, and > 1 indicating synergism, additivity, and antagonism, respectively. For synergistic interactions, CI values between 0.8–0.9 indicate slight synergy, 0.6–0.8 indicate moderate synergy, 0.4–0.6 indicate synergy and those < 0.4 indicate strong synergy. (**D**) CellTiter Glo analysis of OAC cell viability at 72 h post-treatment with ~ IC_30(72 h)_ doses of ALM301 or BV6 alone or in combination. Statistical significance was assessed by an unpaired t-test where NS = not significant, *** = p < 0.001, ** = p < 0.01 and * = P < 0.05. Values are representative of the mean of triplicate experiments ± SEM.

After confirming that BV6-induced depletion of IAPs in OAC cells, the effect of ALM301/BV6 combination treatment on OAC cell viability was assessed. Two-way ANOVA analysis showed that this combination reduced viability with a significant interaction in the OE19, OE33 and FLO1 cells (P < 0.001, P < 0.05 and P < 0.01 respectively, Fig. 4A). This was confirmed as synergistic with CI values < 1 (Fig. 4B). ALM301/BV6 synergy was also confirmed in the OE33 and FLO-1 cells by 2D-clonogenic assay (Fig. 4C).

Cell Titre Glo® was then used to assess IC_30(72 h)_ and IC_50(72 h)_ BV6 doses in the OAC cell line panel and these were combined with ALM301 IC_30(72 h)_ doses (Supplementary Table S2). Compared to either treatment alone, cell viability was significantly reduced following combination treatment in all the OAC cell lines (Fig. 4D). ALM301/BV6 treatment in the OE19, OE33 and FLO-1 and OE19 cells was found to significantly interact by enhancing cytotoxicity (p < 0.001, p0.01 and p < 0.05 respectively; Supplementary Fig. S5).

### Targeting IAP proteins sensitizes resistant OAC cell lines to ALM301

**Fig. 5:**
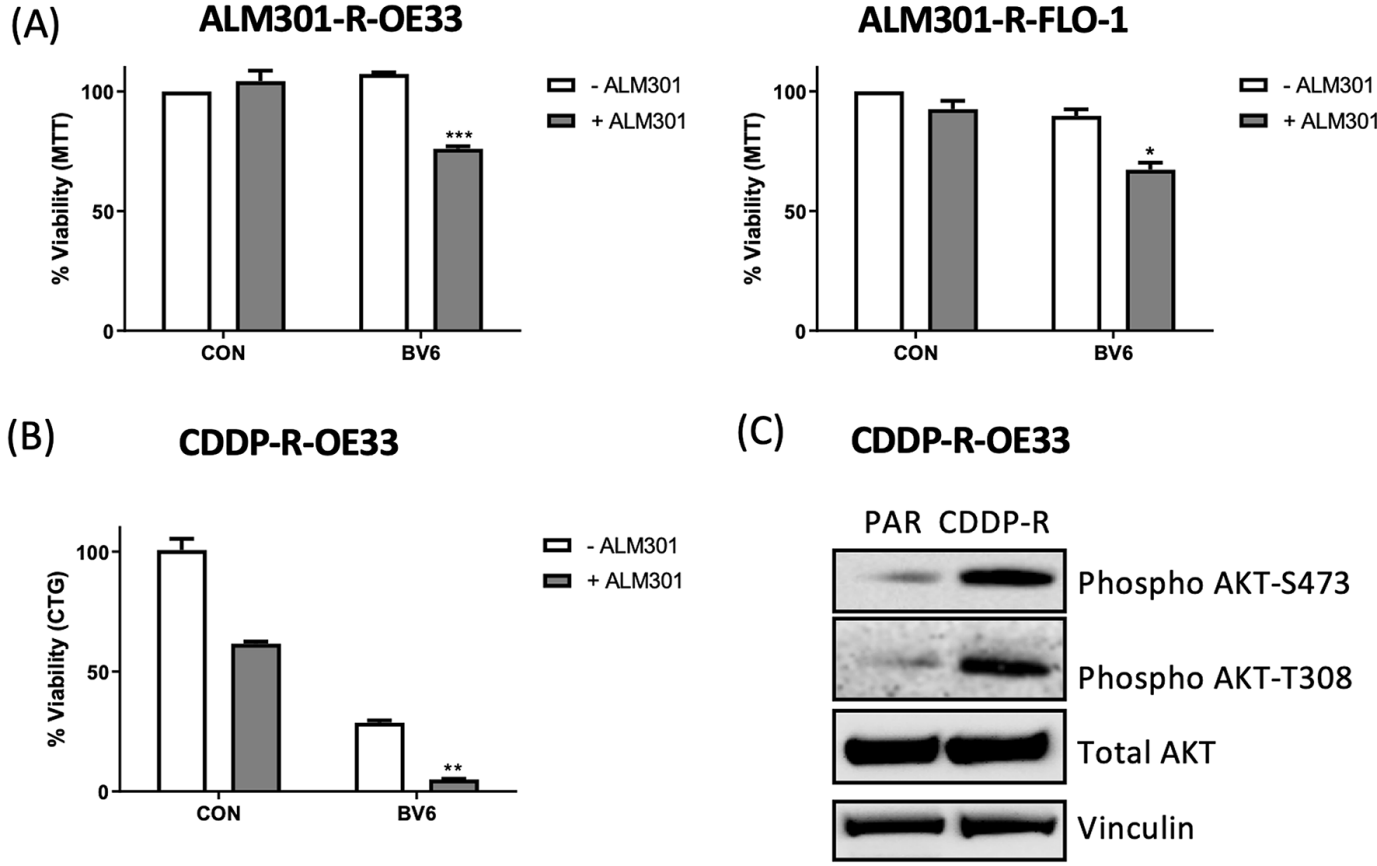
IAP antagonist (BV6) enhances anti-tumour activity of AKT inhibitor (ALM301) in drug-resistant OAC cells. (**A**) MTT analysis of ALM301-R FLO-1 and OE33 cell viability at 72 h post-treatment with parental ~ IC_30(72 h)_ doses of ALM301 alone or in combination with BV6. (**B**) CellTiter Glo analysis of CDDP-R OE33 cell viability at 72 h post-treatment with parental ~ IC_30(72 h)_ doses of ALM301 or BV6 alone or in combination. Statistical significance of the ALM301/BV6 interaction was assessed using a 2-way ANOVA where *** = p < 0.001, ** = p < 0.01 and * = P < 0.05. Values are representative of the mean ± SEM. (**C**) Western blot analysis of constitutive AKT, phospho-AKT-S473 and –T308 in the paired OE33 parental and CDDP-R cells. Vinculin was used as a loading control.

Of importance, BV6 re-sensitized ALM301-R-OE33 and -FLO-1 cells to the parental ~ IC_30(72 h)_ concentrations of ALM301 (Fig. 5A). Cell viability was further reduced when BV6 was combined with ALM301-R ~ IC_30(72 h)_ doses of ALM301 (Supplementary Fig. S6). Targeted therapeutics are often given as a second-line therapy when tumors have become refractory to standard chemotherapy. A CDDP-R-OE33 subline was confirmed as 3.2-fold more resistant than the parental cell line (Table 1). ALM301/BV6 combination treatment significantly interacted to reduce cell viability (p < 0.05; Fig. 5B) in this platinum resistant cell line. Interestingly, the CDDP-R-OE33 had elevated AKT S473- and T308-phosphorylation compared to the OE33 parental cells (Fig. 5C) suggesting that AKT activation may mediate acquired resistance to cisplatin in this OAC cell line.

IAP targeting siRNAs were then used to confirm that the ALM301-sensitizing phenotype was an on-target effect of BV6. Knockdown was confirmed at the protein level (Supplementary Fig. S7A). Sensitization to ALM301 was observed with XIAPsi in all 3 cell lines (Supplementary Fig. S7B); whereas cIAP2si sensitized to ALM301 in the OE33 and FLO-1 cells and BIRC5si sensitized to ALM301 in OE33 and OE19. Interestingly, cIAP1si did not sensitize to ALM301. Further analysis in the OE33 cells showed that cIAP1 knockdown resulted in increased cIAP2 and Survivin expression, highlighting a potential compensatory effect by other IAP family proteins (Supplementary Fig. S7C). Therefore, targeting single IAP family members does not replicate the effect of the IAP antagonist BV6, a compound that simultaneously targets multiple IAP family members. Despite this, these siRNA results do lend support to an on-target ALM301-sensitizing effect of BV6 through the depletion of IAP proteins.

This study uses ALM301 and BV6 as tool compounds for AKT and IAP inhibition respectively. To confirm that ALM301 synergy was not limited to the BV6 compound, other IAP antagonists were used in combination with ALM301 to recapitulate the effect of BV6. ALM301 was combined with the IAP antagonists, AZD5582 and ASTX660. A significant reduction in OAC cell viability was observed following ALM301/AZD5582 combination treatment (P < 0.001) (Supplementary Fig. S7D). Also, colony formation assays showed that ASTX660 was synergistic with ALM301 in the OE33 and FLO-1 cells (Supplementary Fig. S7E). To confirm that synergy was not limited to allosteric AKT inhibition, an OE33 colony formation assay was used to assess the ATP-competitive AKT inhibitor AZD5363 in combination with ASTX660. This resulted in a strong synergistic (CI < 0.4) interaction whereby OE33 colony growth was reduced (Supplementary Fig. S7F).

### Targeting IAP proteins with BV6 synergizes with ALM301 in OAC Organoids

**Fig. 6:**
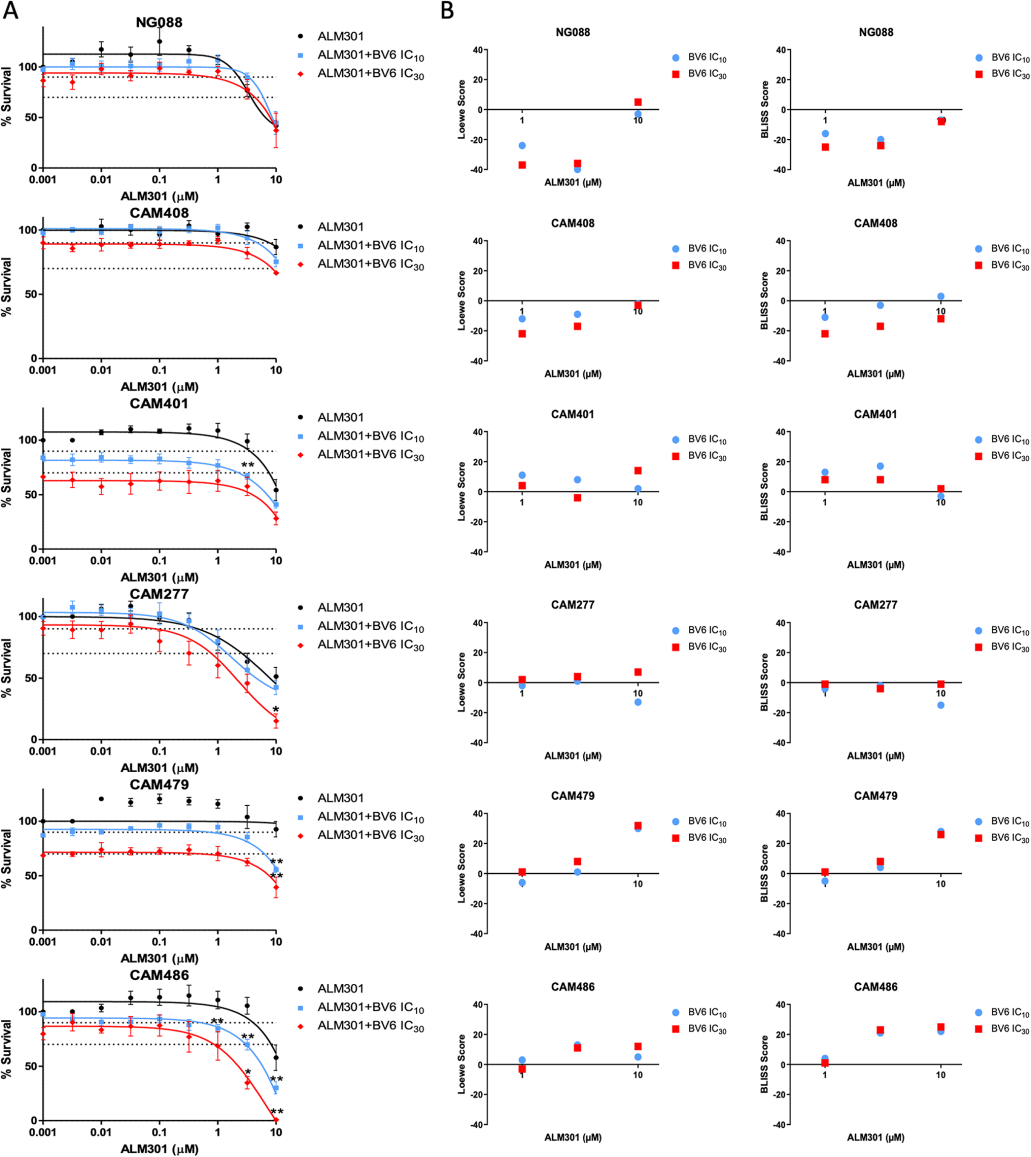
Synergy between IAP antagonist (BV6) and AKT inhibitor (ALM301) in OAC organoid models. (**A**) CellTiter Glo viability of OAC organoids following ALM301 /BV6 combination treatment. Significant interaction by 2-Way ANOVA is represented as P value < 0.05*, 0.01** and 0.001*** (**B**) ALM301/BV6 synergy analysis using Loewe and BLISS methods where positive scores represent synergy, negative scores represent antagonism and scores of 0 represent an additive effect.

OAC organoid cultures can recapitulate the morphology and heterogeneity of OAC tumors and so provide a more accurate representation of OAC biology for target validation. We sought to assess the efficacy of the ALM301/BV6 combination in a panel of OAC organoids established from surgical resection tissue^21^. Five OAC organoids were cultured (CAM277, CAM401, CAM408, CAM468, CAM479) as well as a normal oesophageal columnar mucosa control (NG088). An anchored approach to drug dosing was applied, where a range of doses of ALM301 (1 nM to 10 µM) were used alone or in combination with previously identified ~ IC_10_ and ~ IC_30_ doses of BV6 (Fig. 6A and Supplementary Table S4). Two-way ANOVA analysis identified significant ALM301/BV6 interactions in organoid models CAM401, CAM277, CAM479 and CAM486, where combinations of BV6 (~ IC_10_ or ~ IC_30_ doses) and ALM301 (1, 3 or 10 µM) further reduced organoid viability. BLISS and Loewe additivity analysis showed this to be synergistic, as indicated by positive scores, in the CAM401, CAM479 and CAM486 models (Fig. 6B). ALM301 and BV6 had an additive effect in the CAM277 model, as indicated by scores = 0. ALM301 and BV6 were antagonistic, as indicated by negative scores, in the normal oesophageal organoid, NG088, and OAC organoid CAM408. Of note, CAM408 had the lowest *BIRC2* (cIAP1) and *BIRC5* (Survivin) RNA expression levels of in this organoid panel (Supplementary Table S4).

## Discussion

Hyper-activation of the AKT pathway is associated with resistance to chemotherapy^22^. In this study, we tested the therapeutic potential of AKT inhibition in OAC using the novel allosteric AKT inhibitor, ALM301. We demonstrated that inhibition of AKT synergized with chemotherapy in OAC cell lines in association with down-regulation of the IAP family of proteins. Our study identifies IAPs as mediators of resistance to AKT inhibition and demonstrates the efficacy of combined AKT/IAP inhibition as a novel therapeutic strategy in OAC. The AKT inhibitor, ALM301, and the IAP-targeting SMAC mimetic, BV6, synergized in OAC cells. This drug synergy was also demonstrated with other AKT inhibitor/IAP antagonist combinations and was therefore not limited to the ALM301 and BV6 compounds. This study reports for the first time that AKT inhibition sensitizes OAC cell lines to chemotherapies, 5-FU or CDDP, both of which are used as standard treatment of OAC. AKT inhibition is also reported to enhance the anti-tumor activity of chemotherapies and targeted agents across a range of cancer cell types^6–8^.

CDDP sensitization via AKT inhibition has also been demonstrated in other cancer types^23,24^. In bladder cancer, the AKT inhibitor, MK-2206, potentiated cisplatin-induced apoptosis in T24 and RT112 cell lines and also suppressed RT112 xenografts. In gastric cancer, MK-2206 enhanced cisplatin in AGS cells but not in MKN-45 or MGC-803 cells. Additionally, 5-FU sensitization by AKT inhibition has also been reported in other cancer types^25–27^. In gastric cancer, MK-2206 synergized with 5-FU to reduce SGC-7901 and MKN45 cell viability but only enhanced cytotoxicity in the SGC-7901 cells. A synergistic reduction of cell viability was observed following MK-2206/5-FU co-treatment of lung NCI-H460 and ovarian A2780 cells with increased sub-G1 in the A2780 cells. In colorectal cancer (CRC), miR-587 conferred 5-FU resistance in the HCT116 and GEO cell lines through AKT signaling and this was reversed by MK-2206 treatment. In agreement with our OAC findings, these reports demonstrate the chemo-sensitizing phenotype of AKT inhibition but also highlight the context-dependent nature of this effect.

Elucidating the mechanisms that promote AKT inhibitor/chemotherapy synergy could inform a biomarker strategy for patient stratification as well as identify more effective combination therapeutics. The IAP protein family were originally discovered to negatively regulate programmed cell death through caspase inhibition^28^. However, it is now known that IAPs impact cell survival through other mechanisms such as cytokine signaling, NF-kB signaling, cell-extracellular matrix interactions, cell cycle, migration, immunity and MYC activity^29–31^. They are frequently overexpressed in cancer, including OAC, contributing to chemo-resistance and correlating with a poorer patient outcome^32–34^. In particular, high XIAP expression is predictive of a poor prognosis in node negative and highly T-cell infiltrated OAC tumors as well as identifying a subset of responders to neo-adjuvant therapy who are prone to relapse^35^ Whilst XIAP is well established as an anti-apoptotic factor enabling cancer cells to resist cytotoxic therapy, recent evidence has identified XIAP as an important component of tumor-associated inflammation^36–38^. Our study found that members of the IAP protein family, cIAP1, cIAP2, XIAP and Survivin, were acutely down-regulated by chemotherapy in OAC cell line settings that demonstrated synergy with ALM301. In addition, IAP proteins were upregulated in OAC cell lines with acquired ALM301 resistance. Moreover, high XIAP protein expression was associated with intrinsic resistance to ALM301. These findings lend support for high XIAP protein expression as a biomarker of resistance to AKT inhibition in OAC. However, further analysis with a greater sample set would be required to validate this.

In the OAC cell line panel, neither AKT expression/ phosphorylation nor the expression of the endogenous pathway inhibitor, PTEN, conferred sensitivity to ALM301. This is in contrast to a previous report in a panel of 100 cancer cell lines comprising of breast, cervical, colon, endometrial, ovarian, pancreatic and prostate cell lines, where high basal pAKT-S473 correlated with sensitivity to the ATP-competitive inhibitor, Ipatasertib (GDC-0068)^20^. However, OAC cell lines were not included in the Ipatasertib study and these conflicting results may reflect the biology of OAC and/or the molecular class differences between the allosteric, ALM301, and the ATP competitive, Ipatasertib, AKT inhibitors. The lack of a predictive biomarker in OAC was highlighted in a Phase II trial of Ipatasertib compared to placebo as first-line therapy in metastatic gastric and gastro-oesophageal junction adenocarcinoma in combination with oxaliplatin and 5-FU chemotherapy (FOLFOX). Ipatasertib failed to show an improvement in PFS and OS over chemotherapy alone in the intention-to-treat population and also in a biomarker-selected (PTEN-low or PI3K/AKT pathway activated) tumours^39^.

Targeting IAPs has become an increasingly attractive strategy to re-sensitize cancer cells to chemotherapies, targeted therapies and radiotherapy^28,40–42^. Small-pharmacological molecules that mimic the N-terminal IAP binding motif (IBM) of the endogenous IAP protein antagonist, second mitochondria-derived activator of caspases (SMAC), have been developed in recent years. BV6 depleted cIAP1, cIAP2 and XIAP in the OAC cell lines and this was found to synergize with ALM301 and also re-sensitize OAC cell lines with acquired resistance to ALM301. IAP antagonists trigger rapid proteasomal degradation of c-IAP1 and c-IAP2 while antagonizing XIAP through direct binding. For BV6, XIAP reduction has been attributed to caspase-mediated cleavage during ongoing apoptosis^43^. However, BV6-induced XIAP depletion in OAC cells was independent of caspase activity and this is in agreement with a previous report in PI3K mutant CRC HCT116 cells^44^. Of note, the OE33, FLO-1 and OE19 cells also harbor PI3K mutations.

Co-operation of an IAP antagonist with inhibitors of the PI3K/AKT/mTOR pathway to enhance anti-tumour activity has been previously reported. Blocking NF-κB and AKT by Hsp90 inhibition sensitized lung adenocarcinoma cells, H23, and hepatocellular carcinoma cells, HepG2, to IAP antagonists resulting in synergistic cancer cell death^45^. Inhibition of the NF-κB or AKT pathway was also found to sensitize resistant nasopharyngeal carcinoma (NPC) cells to the IAP antagonist, APG-1387^46^. A PI3K inhibitor, LY294002, was reported to synergize with an IAP antagonist in non-small cell lung carcinoma (NSCLC) H1299 cells via suppression of cIAP2 induction^47^. Whilst the present study reports BV6/ALM301 synergy in OAC cell lines, it does not address the mechanism and this requires further study.

The identification of this synergistic drug combination of an allosteric AKT inhibitor and IAP antagonist provides a promising novel therapeutic strategy for OAC. Importantly, this drug combination was effective in a CDDP-resistant OAC cell line and an OAC organoid CAM401 derived from a patient with a tumor regression grade (TRG) of 5, indicating no pathological response to neoadjuvant therapy incorporating Cisplatin and 5-FU^21^. This supports a potential means for overcoming chemo-resistance in OAC patients. Several AKT inhibitors and IAP antagonists have already progressed through clinical trials providing some indication of the potential treatment characteristics of this combination. MK2206, a potent allosteric AKT inhibitor, and AZD5363, a selective ATP-competitive pan-AKT kinase inhibitor, are both administered orally and despite their differing mechanisms of action both can cause a maculo-papular rash, nausea, vomiting, diarrhea, fatigue and hyperglycemia^48,49^. The most common toxicities of the current, clinically advanced IAP antagonists, Debio1143 and ASTX660, are also gastrointestinal in nature with both Debio1143 and MK2206 capable of causing elevated alanine aminotransferase levels^50,51^. The overlapping toxicities of these two classes of targeted agents may make clinical development of suitable dosing schedules challenging.

## Conclusions

Our findings show the utility of the novel allosteric AKT inhibitor, ALM301, as a potential OAC therapeutic. This study reports for the first time that AKT inhibition can sensitize OAC cell lines to Cisplatin and 5-FU which are used in the chemotherapy treatment of OAC. We also identify a novel synergistic drug combination of AKT and IAP family inhibition, providing strong pre-clinical evidence and justification for further investigation of this treatment strategy in OAC.

## Methods

### Cell culture

All OAC cell lines were obtained from Public Health England. OE19, OE33, SKGT4, ESO26, ESO51, OACP4C and OACM5.1C were maintained in RPMI-1640 medium supplemented with 10% Foetal Bovine Serum (FBS), 50 μg/mL penicillin–streptomycin, 2 mmol/L l-glutamine, and 1 mmol/L sodium pyruvate at 37 °C in a humidified atmosphere containing 5% CO_2_. The KYAE-1 cell line was maintained in 50% RPMI-1640, 50% Hams F12 supplemented with 10% Foetal Bovine Serum (FBS), 50 μg/mL penicillin–streptomycin, 2 mmol/L l-glutamine, and 1 mmol/L sodium pyruvate at 37 °C in a humidified atmosphere containing 5% CO_2_. The FLO-1 cell line was maintained in DMEM supplemented with 10% Foetal Bovine Serum (FBS), 50 μg/mL penicillin–streptomycin, 2 mmol/L l-glutamine, and 1 mmol/L sodium pyruvate at 37 °C in a humidified atmosphere containing 5% CO_2_. All lines were routinely examined for mycoplasma contamination.

### Cell viability analysis

Cell viability was determined at 72 h post-treatment using MTT or CellTiter Glo assays. Briefly, exponentially growing cells were seeded into 96-well plates, treated at 18 h post-seeding and viability was measured at 72 h post-treatment. GraphPad software (Prism version 8, https://www.graphpad.com/). was used generate dose–response curves and calculate the ~ IC_30(72 h)_ and ~ IC_50(72 h)_ drug doses.

### Colony formation assay

Colony formation assays were used to measure viability following long-term drug treatment. Briefly, exponentially growing OAC cells were seeded into 6-well plates at 400 cells per well. At 18 h post-seeding, cells were treated with either ALM301, 5-FU, CDDP, BV6 alone or in combination. The cells were incubated for ~ 10–14 days post-treatment prior to fixation in methanol and staining with crystal violet. Colonies were counted using a GelCount colony counter (Oxford Optronix, Abingdon UK). GraphPad software (Prism version 8, https://www.graphpad.com/). was used generate dose–response curves and calculate the ~ IC_30_ and ~ IC_50_ drug doses.

### Flow cytometric analysis

DNA content of harvested cells was evaluated after propidium iodide (PI) staining of cells using the EPICS XL Flow Cytometer (Coulter, Miami, FL, USA). The sub-G1 population was determined by evaluating the percentage of cells with DNA content < 2 N. Cytotoxicity was confirmed by Annexin V/PI analysis. Cells were harvested and analyzed according to the manufacturer’s instructions (BD Biosciences, San Jose CA).

### Proliferation assay by cell count

Cells were seeded at 10^5^ per cells/well into a 6-well plate and treated with ALM301 alone or in combination with chemotherapy. At 24, 48 and 72 h post-treatment, the cells were re-suspended and Trypan Blue exclusion was used to evaluate viable cells. Cells were counted using a Countess (ThermoFisher Scientific, UK) and the mean count ± the standard error of the mean (SEM) from 3 independent experiments was calculated.

### Western blotting

Western blot analysis was carried out as previously described^52^ using antibodies targeting phospho-(S473)-Akt (Cell Signaling Technology Cat# 4060, RRID:AB_2315049), phospho-(T308)-Akt (Cell Signaling Technology Cat# 4056, RRID:AB_331163), pan- Akt (Cell Signaling Technology Cat# 2920, RRID:AB_1147620), PTEN (Cell Signaling Technology Cat# 9559, RRID:AB_390810), PARP (Cell Signaling Technology Cat# 9532, RRID:AB_659884), phospho-γ-H2AX (Cell Signaling Technology Cat# 9718, RRID:AB_2118009), cIAP1 (Cell Signaling Technology Cat# 7065, RRID:AB_10890862), cIAP2 (Cell Signaling Technology Cat# 3130, RRID:AB_10693298), XIAP (Cell Signaling Technology Cat# 2045, RRID:AB_2214866) and Survivin (Cell Signaling Technology Cat# 2808, RRID:AB_2063948). Mouse monoclonal antibodies were used in conjunction with anti-mouse IgG, HRP-linked Antibody (Cell Signaling Technology Cat# 7076, RRID:AB_330924). Rabbit polyclonal antibodies were used in conjunction with with anti-rabbit IgG, HRP-linked Antibody (Cell Signaling Technology Cat# 7074, RRID:AB_2099233). Equal loading was assessed using vinculin (Abcam Cat# ab129002, RRID:AB_11144129) or β-actin (Cell Signaling Technology Cat# 3700, RRID:AB_2242334) targeting antibodies.

### Q-PCR

Total RNA was isolated using RNA STAT-60 reagent according to the manufacturer’s instructions (AMS Biotechnology, Abingdon, UK). Reverse transcription was carried out using Transcriptor first strand cDNA synthesis kit (Roche Diagnostics) according to the manufacturer’s instructions. Quantitative reverse transcription-PCR (RT- PCR) amplification was carried out in a final volume of 10μL containing 5μL of LightCycler 480 Probes Master (Roche Diagnostics) and 1μL of RealTime ready Assay (Roche Diagnostics) 1.5μL of PCR grade H_2_O (Roche Diagnostics) and 2.5 μl of cDNA using a Light Cycler® 480 II (Roche Diagnostics) according to the manufacturer’s protocols.

### Organoid derivation and culture

Organoids were established and cultured as previously described^21^. Experiments were performed in triplicate and AUC and IC_50_ values are the mean across all replicates. The drug combinations were conducted using an anchored approach, whereby the dose of BV6 was kept constant at IC_10_ and IC_30_, calculated individually for OACh organoid, and a 9-point dilution series of ALM301 was added. Cell viability was measured using CellTiter-Glo® 3D Cell assay (Promega) following 6 days of drug incubation.

### Synergy analysis

Synergy in the 2D cell line assays was assessed by Combination index (CI). Compusyn version 1 software (https://compusyn.software.informer.com) used to calculate CI values according to the method of Chou and Talalay^53^. CI values < 1, 1, and > 1 indicate synergy, additivity, and antagonism, respectively. For synergistic interactions, CI values between 0.8 and 0.9 indicate weak synergy, 0.4 and 0.8 indicate moderate synergy, and < 0.4 indicate strong synergy^54^.

Synergy in the organoid assays was assessed using the Loewe additivity and BLISS scores where negative scores indicate antagonism and positive scores indicate synergy.

### Statistical analysis

All t tests and 2-way ANOVAs were calculated using the GraphPad software (Prism version 8, https://www.graphpad.com/). Specifically, t tests were unpaired, 2-tailed using 95% confidence intervals. Two-way ANOVA was calculated using 95% confidence intervals and a Bonferroni post-hoc test.

### siRNA transfection

All siRNAs were supplied by Qiagen (Crawley, UK). All Stars negative control (NC) and All Stars Death control were used as a non-targeting and positive control, respectively. OAC cells were reverse-transfected using siRNA in Opti-MEM reduced-serum media to a final concentration of 10 nM and HiPerFect transfection reagent (Qiagen, Crawley, UK). Knock-down of the targeted gene was assessed at 72 h post-transfection. Where transfection was combined with drug treatment, cells were transfected for 24 h prior to drug.

### Generation of drug resistant OAC cell lines

OE33 and FLO-1 cell lines were continuously exposed to escalating concentrations of ALM301, starting at ~ IC_10(72 h)_ doses, over a period of ~ 3 months. Similarly, OE33 cells were continuously exposed to increasing concentrations of CDDP, starting at ~ IC_10(72 h)_ doses, over a period of ~ 3 months. Dose response curves from MTT assays were applied to calculate ~ IC_30(72 h)_ doses.

### Transcriptomic analysis of paired OAC biopsies and resection specimens

Transcriptomic profiling of 17 pre-treatment FFPE OAC endoscopic biopsies and 17 surgical resection specimens was performed using the Almac Diagnostics Xcel™ array (Almac, Craigavon, UK) as previously described^55^. All patients were enrolled in the randomised phase II dose expansion component of the DEBIOC clinical trial (EudraCT 2011-003169-13) as previously described^56^. The DEBIOC study was conducted in accordance with the International Conference of Harmonisation of Good Clinical Practice and the Declaration of Helsinki. Ethical approval was provided by the National Research Ethics Service Committee South Central (Oxford, ethics no. 12/SC/0090) and all participants provided written informed consent. Patients were randomised 2:1 to receive either Oxaliplatin (130 mg/m^2^ IV over 2 h on day one of every cycle) and Capecitabine (1250 mg/m^2^ bd) (Xelox) + AZD8931 (20 mg bd 4d on/3d off) for two 21-day cycles, or Xelox alone as neoadjuvant treatment prior to oesophagectomy at 4 UK centres (Belfast, Leicester, Oxford and Bristol). 

## Supplementary Information

Supplementary Information 1.

Supplementary Information 2.

Supplementary Information 3.

## Data Availability

The datasets generated or analysed during the current study are available from the corresponding author on reasonable request.
