# Supplementary material for: Inhibition of AKT enhances chemotherapy efficacy and synergistically interacts with targeting of the Inhibitor of apoptosis proteins in oesophageal adenocarcinoma
Source: Sci Rep. 2024 Dec 30;14:32121. doi: 10.1038/s41598-024-83912-4 (PMC11686190; doi:10.1038/s41598-024-83912-4)
Supplement: Supplementary file 2 — Supplementary Information 2. [file 41598_2024_83912_MOESM2_ESM.pdf]

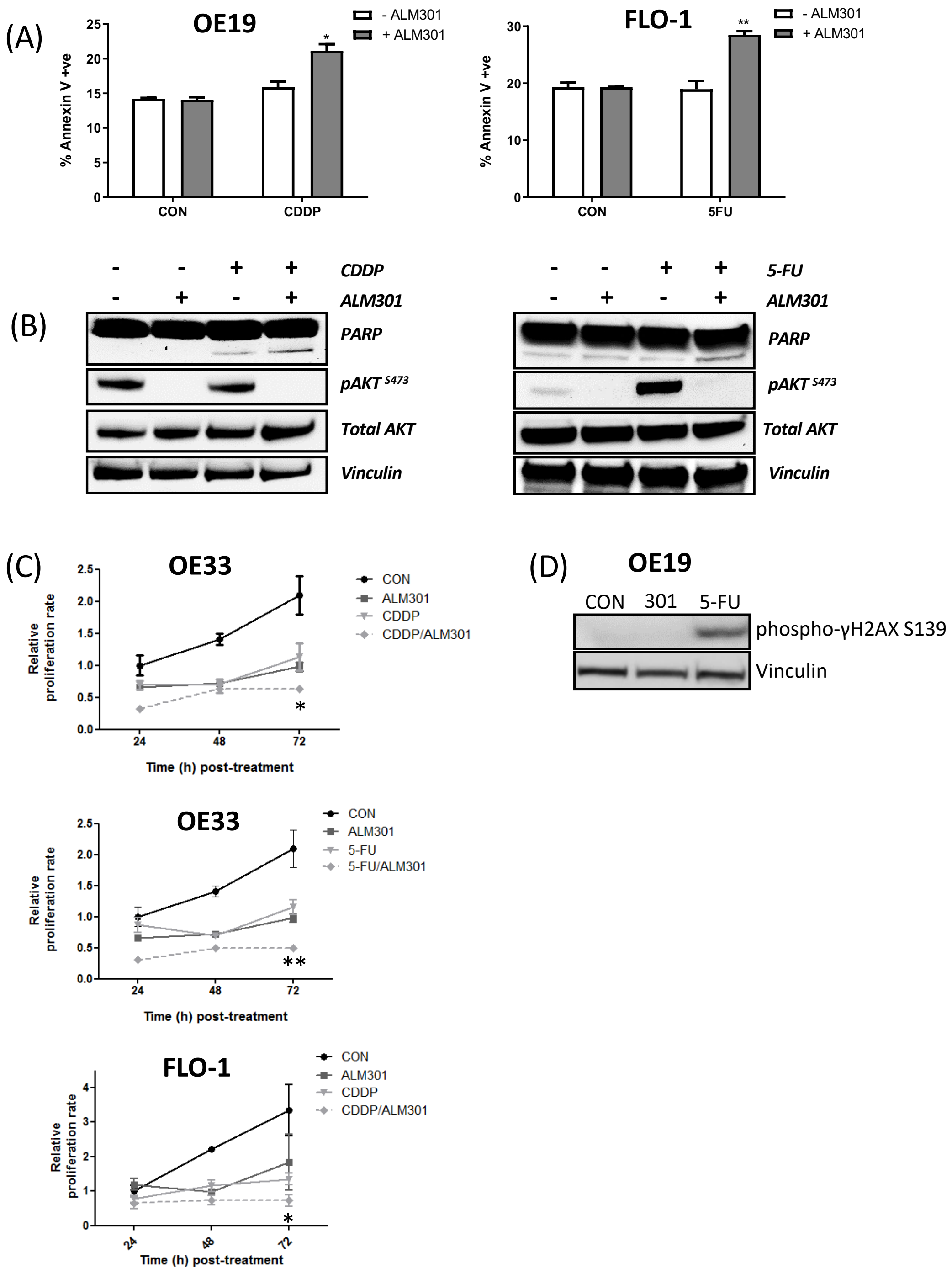


**Supplementary Figure S1 . ALM301/chemotherapy synergy is attributable to cytoxicity or cytostasis** (A) Flow cytometric analysis of the Annexin V positive OAC cell population at 72h post-treatment with ~$IC_{30(72h)}$ doses of ALM301 or chemotherapy alone or in combination. Statistical significance of ALM301/chemo interaction was assessed by using a 2-way ANOVA where *** = p<0.001, ** = p<0.01 and * = P<0.05. Data is representative of the mean ± standard error of the mean. (B) Western blot analysis of cleaved PARP at 72h post-treatment with ~$IC_{30(72h)}$ doses of ALM301 or chemotherapy alone or in combination. Vinculin was used a loading control. (C) Viable OAC cells were counted at 24, 48 and 72h post treatment with ~$IC_{30(72h)}$ doses of ALM301 or chemotherapy alone or in combination. Proliferation rates were calculated by normalising to the control cell count at 24h. Statistical significance of ALM301/chemotherapy combination compared to either treatment alone was assessed by using an unpaired t-test where *** = p<0.001, ** = p<0.01 and * = P<0.05. Data is representative of the mean ± standard error of the mean. (D) Western blot analysis of phospho-γ-H2AX to confirm 5-FU-induced DNA damage in OE19 cells at 72h post-treatment with ~$IC30_{(72h).}$

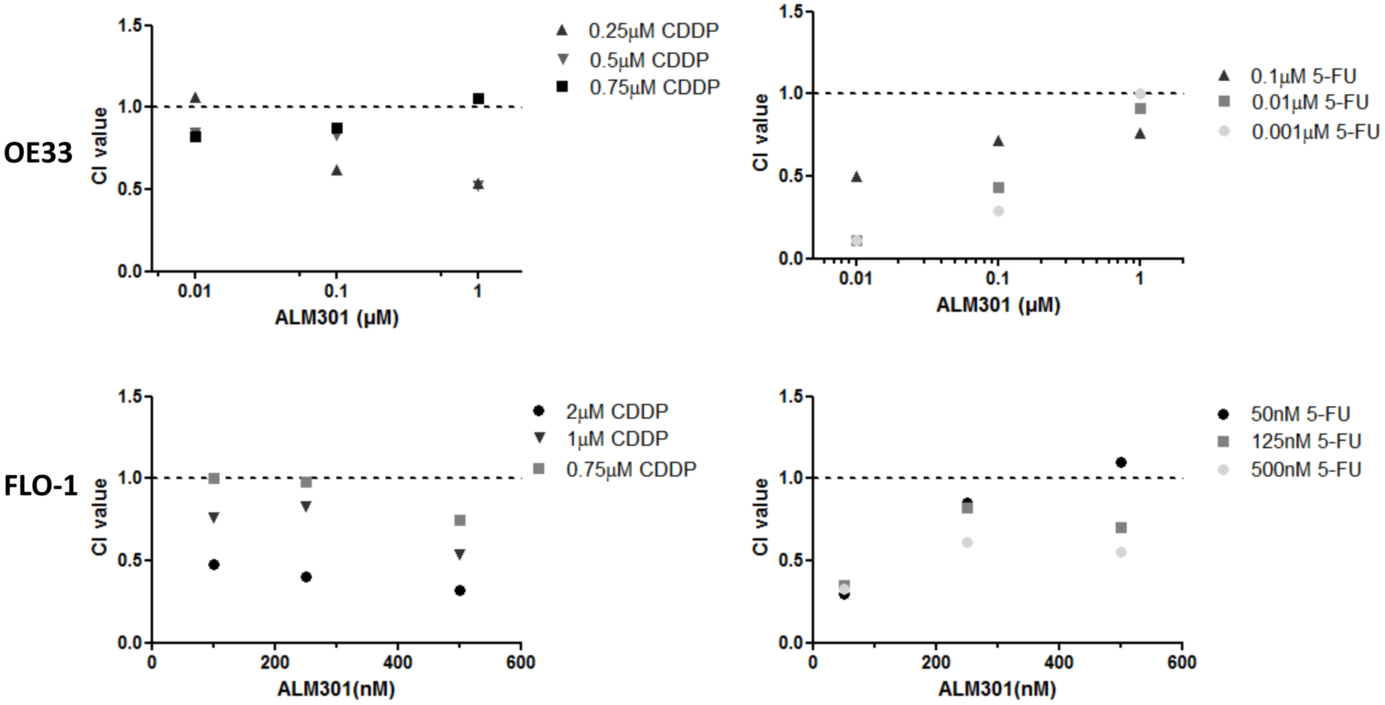


**Supplementary Figure S2.** Colony formation assays were used to assess OAC cell viability at ~10-14 days post-treatment with ALM301/chemotherapy combinations using drug doses of ~$IC_{30(72h)}$ or less. To evaluate the interaction between chemotherapy and ALM301, the method of Chou and Talalay was used to calculate combination index (CI) values. CI values <1, =1, and >1 indicating synergism, additivity, and antagonism, respectively. For synergistic interactions, CI values between 0.8-0.9 indicate slight synergy, 0.6-0.8 indicate moderate synergy, 0.4-0.6 indicate synergy and those <0.4 indicate strong synergy.

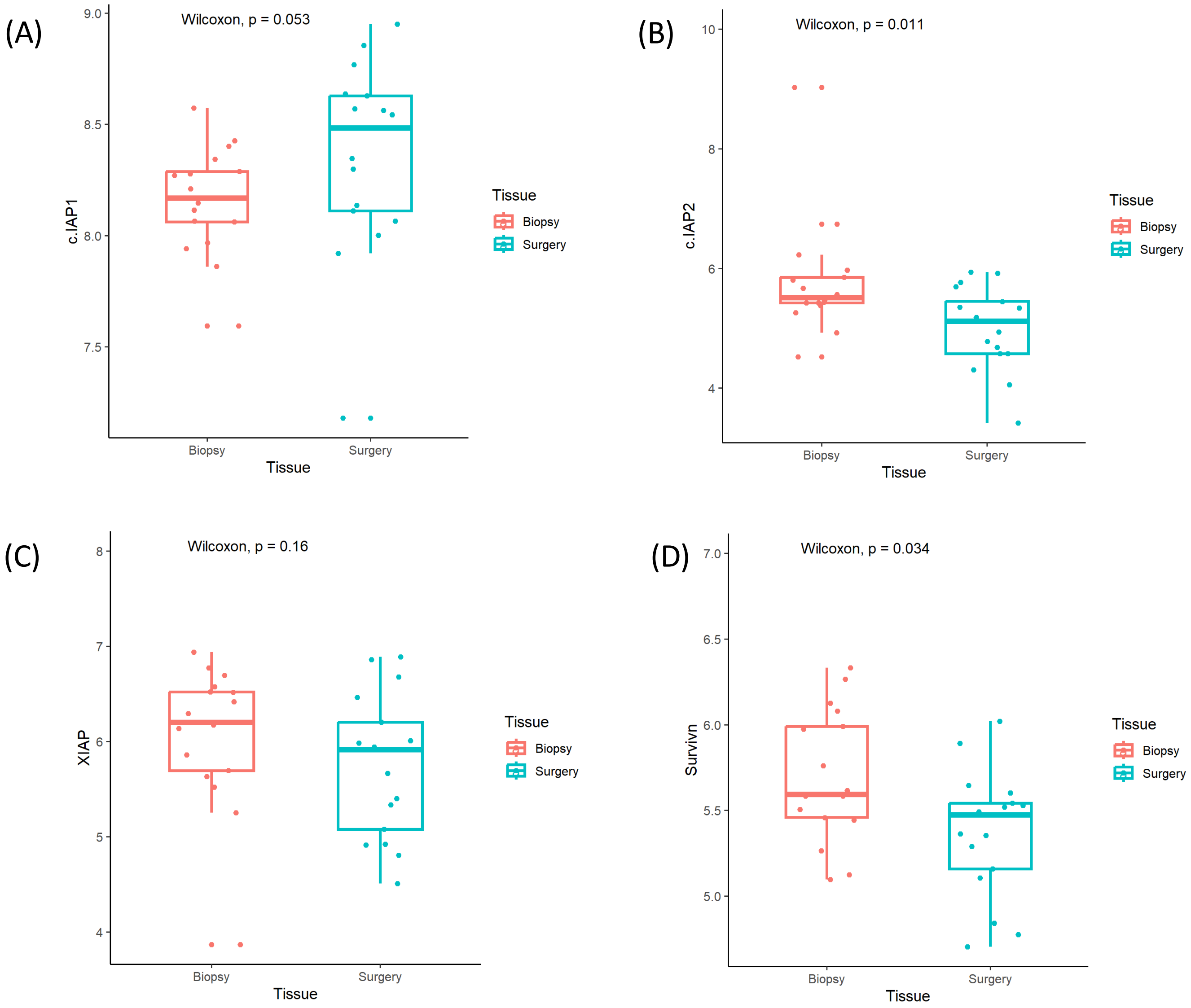


**Supplementary Figure S3.** Boxplots comparing gene expression of IAPs using Wilcoxon signed-rank testing between n=17 paired biopsies and resection specimens demonstrated that neoadjuvant therapy induced changes in expression of (A) cIAP1 (p= 0.053), (B) cIAP2 (p= 0.011), (C) XAIP (p= 0.16) and (D) Survivin (p =0.034).

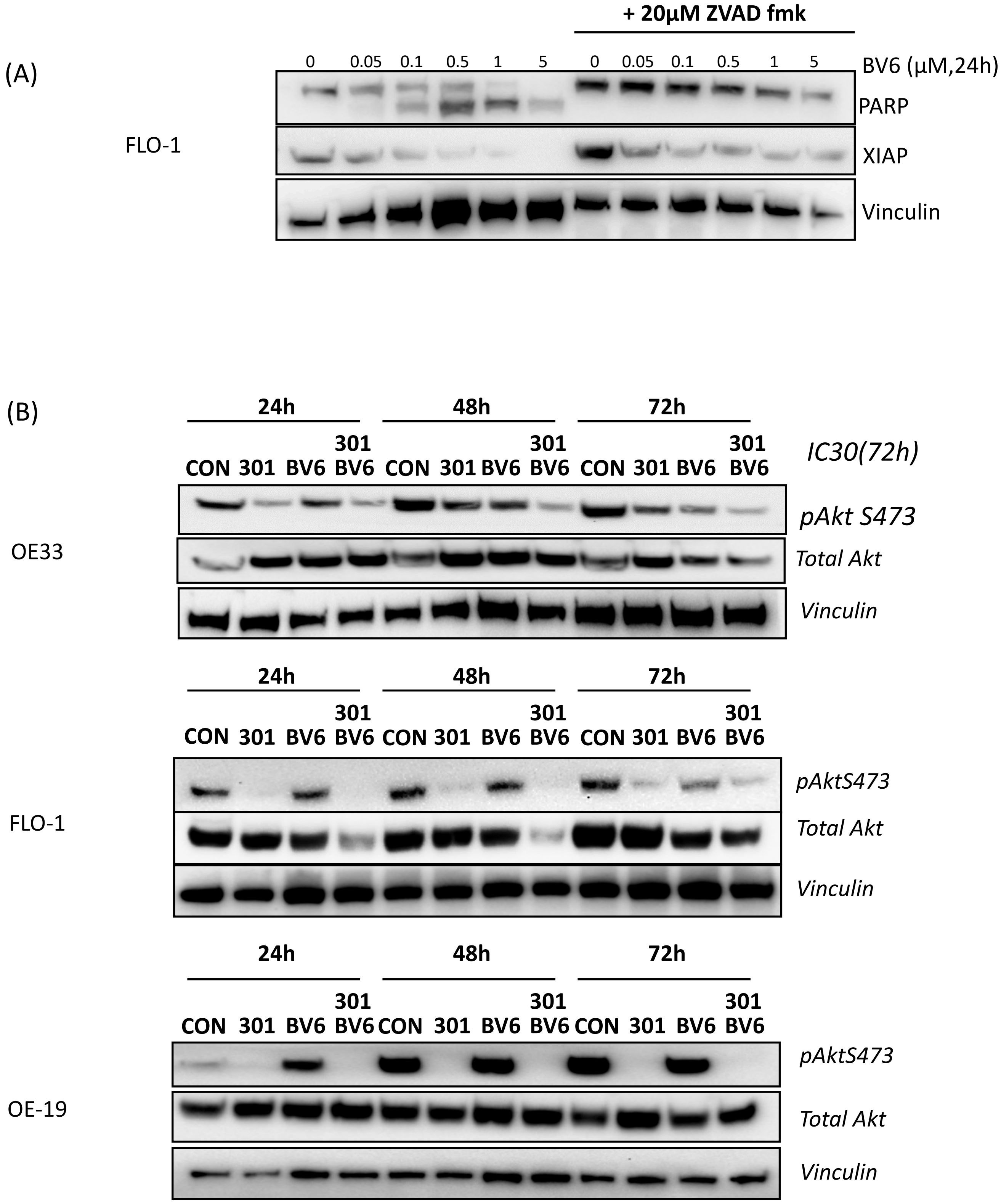


**Supplementary Figure S4**. (A) Western blot analysis of cleaved PARP and XIAP protein levels following 24h treatment with BV6 ± pan caspase inhibitor, ZVAD-fmk. Vinculin was used as a loading control.

(B) Western blot analysis of a 72-hour time course showing phospho-Akt-S473 levels following treatment of OE33, FLO-1 and OE19 cells with ~$IC30_{(72h)}$ doses of BV6 alone or in combination with 1µM ALM301. Vinculin was used as a loading control.

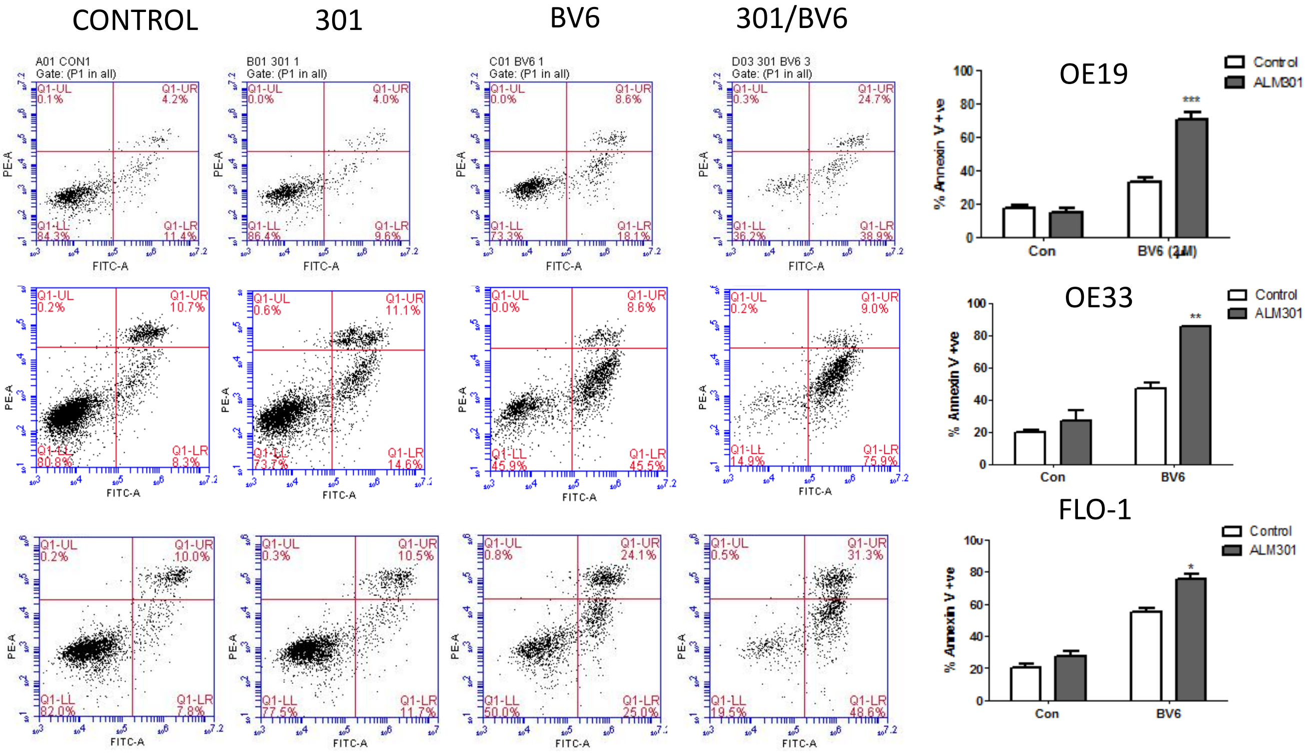


**Supplementary Figure S5.** Flow cytometric analysis of the Annexin V/PI positive EAC cell population at 72h post-treatment with ~$IC_{30(72h)}$ doses of ALM301 or BV6 alone, or in combination. Statistical significance of the ALM301/BV6 interaction was assessed using a 2-way ANOVA where *** = p<0.001, ** = p<0.01 and * = P<0.05. Values are representative of the mean ± SEM.

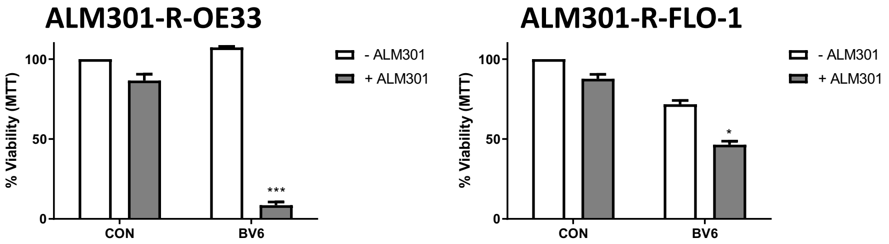


**Supplementary Figure S6.** MTT analysis of ALM301-R FLO-1 and OE33 cell viability at 72h post-treatment with ALM301-R ~$IC_{30(72h)}$ doses of ALM301 alone or in combination with ~$IC_{30(72h)}$ dose BV6. Statistical significance of the ALM301/BV6 interaction was assessed using a 2-way ANOVA where *** = $p<0.001$, ** = $p<0.01$ and * = $P<0.05$. Values are representative of the mean ± SEM.

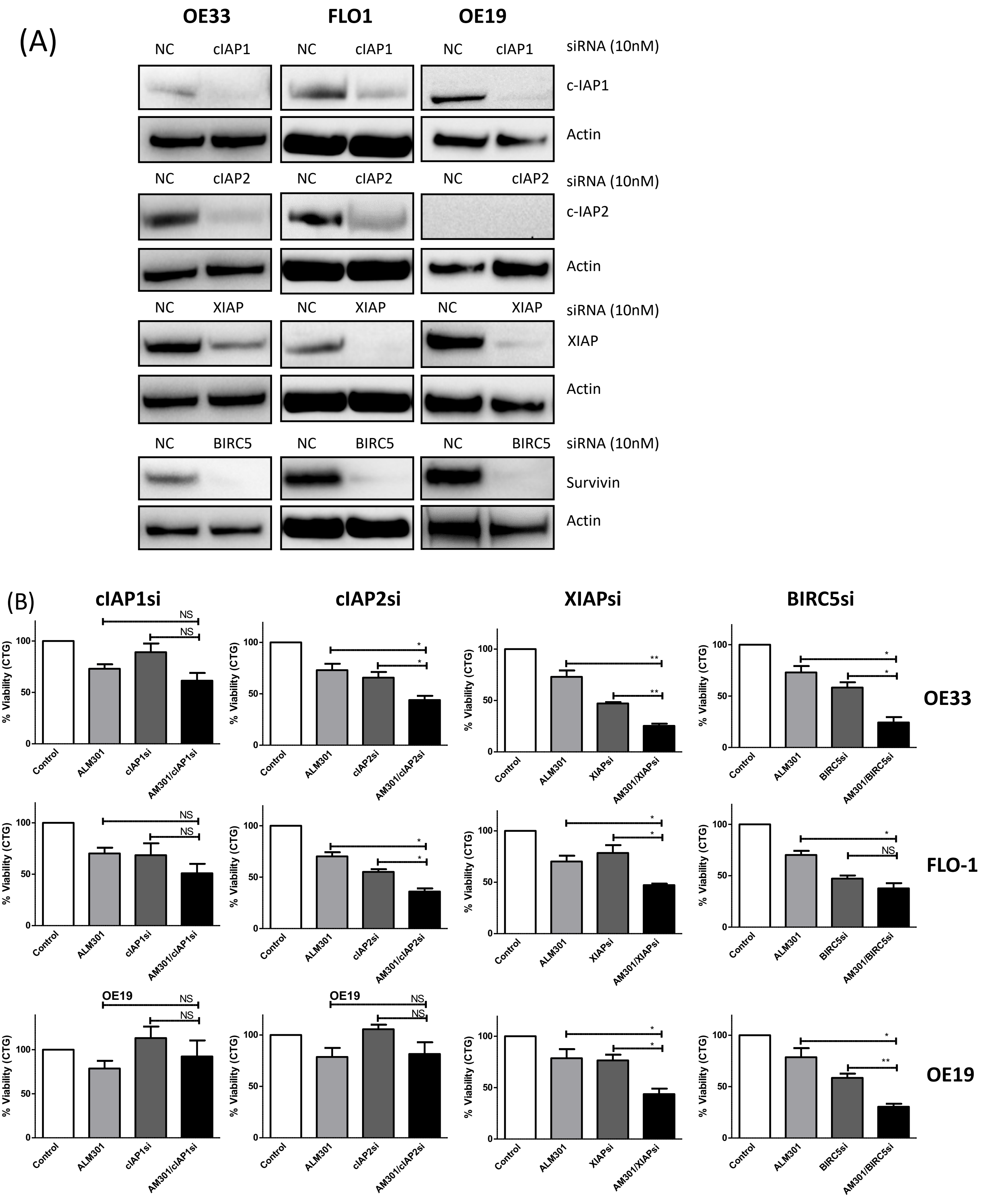


**Supplementary Figure S7.** (A) Confirmation of IAP knock-down in OE33, FLO-1 and OE19 cells. Western blot analysis of cIAP1, cIAP2, XIAP and Survivin following 72h treatment with IAP targeting siRNA. (B) Cell Titre Glo® analysis of OAC cell viability at 72h post-treatment with cIAP1, cIAP2, XIAP or BIRC5-targeting siRNA. Statistical significance was assessed using an unpaired t-test where *** = p<0.001, ** = p<0.01 and * = P<0.05. Values are representative of the mean ± SEM.

(C)

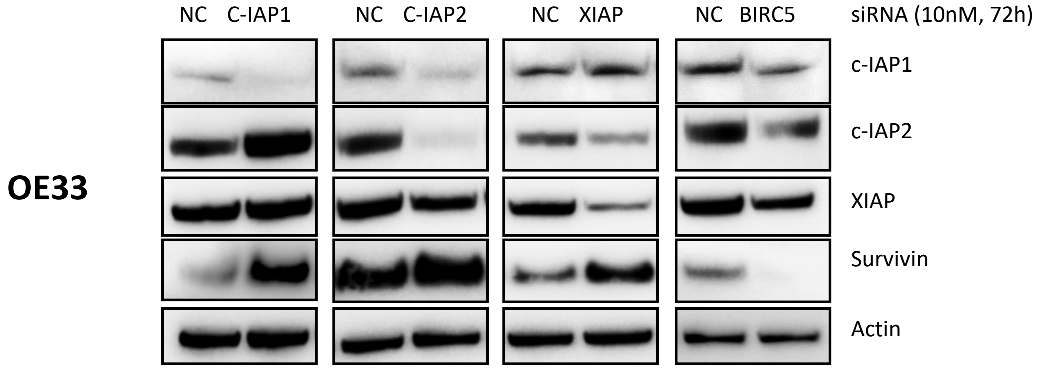


(D)

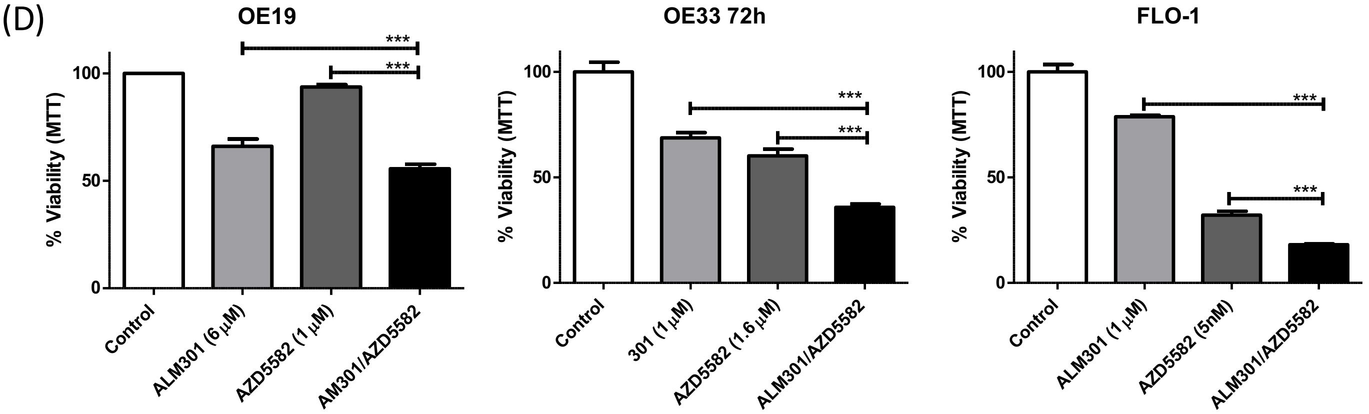


(E)

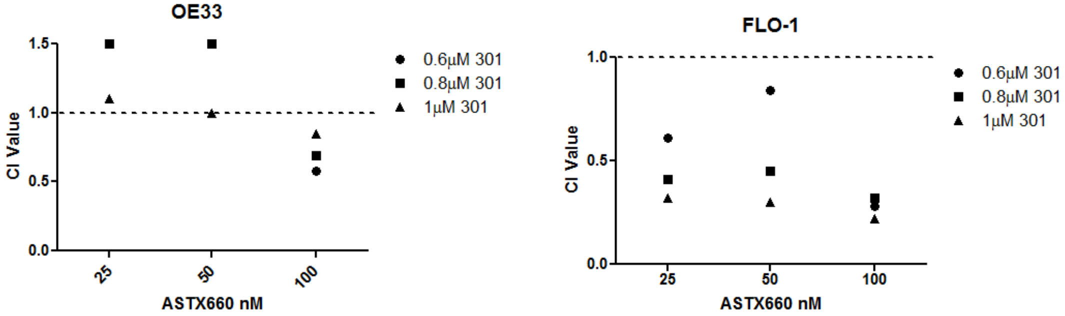


**Supplementary Figure S7.** (C) Western blot analysis of IAP proteins in OE33 cells following 72h knock-down with IAP-targeted siRNA. Actin was used as a loading control. (D) MTT analysis of OAC cell viability at 72h post-treatment with ~$IC_{30(72h)}$ doses of ALM301 or AZD5582 alone or in combination. Statistical significance was assessed by an unpaired t-test where *** = p<0.001, ** = p<0.01 and * = P<0.05. Values are representative of the mean ± SEM. (E) CI values derived from OE33 and FLO-1 colony formation assays following ALM301 and ASTX660 combination treatment.

(F)

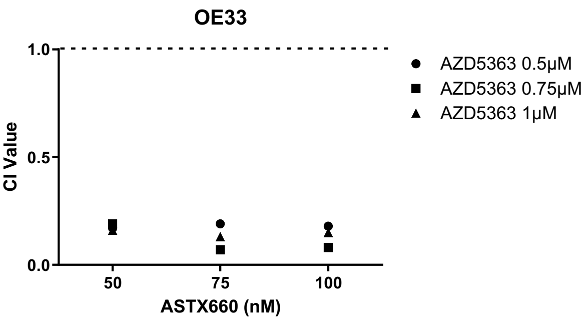


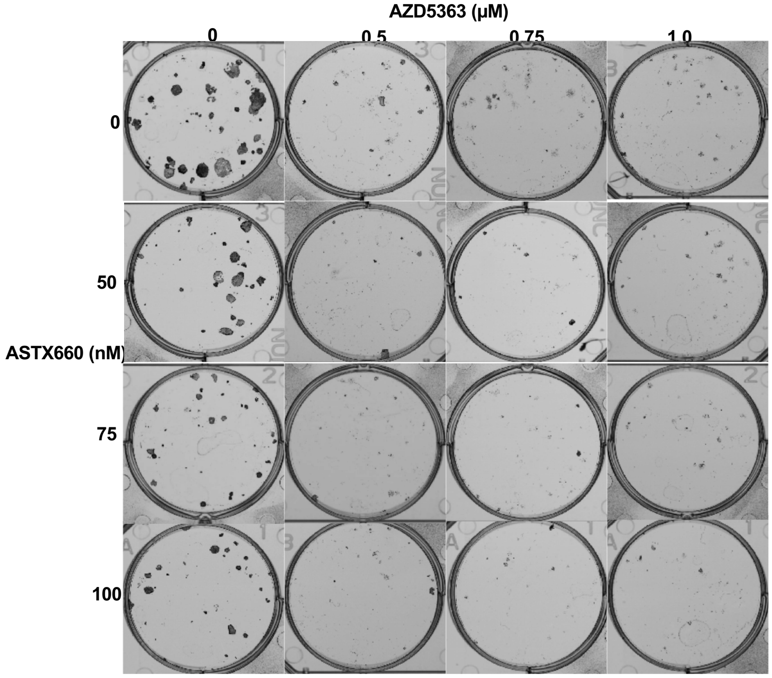


**Supplementary Figure S7**. (F) Colony formation assays were used to assess OE33 cell viability at ~10-14 days post-treatment with AKTi AZD5363 and IAPi ASTX660 combinations using drug doses of ~$IC_{30(72h)}$ or less. To evaluate the interaction, the method of Chou and Talalay was used to calculate combination index (CI) values. CI values <1, =1, and >1 indicating synergism, additivity, and antagonism, respectively. For synergistic interactions, CI values between 0.8-0.9 indicate slight synergy, 0.6-0.8 indicate moderate synergy, 0.4-0.6 indicate synergy and those <0.4 indicate strong synergy.

# SUPPLEMENTARY TABLES

**Supplementary Table S1.** MTT ~$IC_{30(72h)}$ for ALM301, 5-FU and CDDP in OE19, OE33 and FLO1 OAC cells

| Cell Line | ALM301 ~$IC_{30(72h)}$ (µM) | 5-FU ~$IC_{30(72h)}$ (µM) | CDDP ~$IC_{30(72h)}$ (µM) |
|---|---|---|---|
| **OE19** | 6.4 | 6.3 | 12 |
| **OE33** | 1.1 | 0.4 | 1.5 |
| **FLO-1** | 1.5 | 1.1 | 4 |

**Supplementary Table S2.** ALM301 and BV6 sensitivity data in OAC cell lines. Viability was measured by Cell Titre Glo ® assay at 72h post-treatment and ~IC30(72h) and ~IC50(72h) doses were calculated using dose-response curves.

| Cell Line | CellTitre-Glo® ALM301 ~$IC_{30(72h)}$ (µM) | CellTitre-Glo® ALM301 ~$IC_{50(72h)}$ (µM) | CellTitre-Glo® BV6 ~$IC_{30(72h)}$ (µM) | CellTitre-Glo® BV6 ~$IC_{50(72h)}$ (µM) |
|---|---|---|---|---|
| **KYAE1** | 0.8 | 1.2 | 0.17 | 0.39 |
| **OE19** | 6 | 10.3 | 0.9 | 2.2 |
| **OACM5C1** | 1 | 1.8 | 1.3 | 3 |
| **ESO26** | 0.8 | 2.6 | 0.15 | 0.34 |
| **SKGT4** | 1 | 3.6 | 2.6 | 6.2 |
| **OE33** | 1 | 1.6 | 2.4 | 5.8 |
| **ESO51** | 10 | 32.3 | 1.3 | 3.1 |
| **OACP4C** | 1 | 1.4 | 1.2 | 2.8 |
| **FLO-1** | 1 | 1.7 | 0.1 | 0.26 |

**Supplementary Table S3.** Densitometry values, relative to a vinculin loading control, of basal protein expression or phosphorylation in OAC cell lines. Values are represented as normalised to the median.

| Cell Line | pAKT S473 | pAKT T308 | Dual pAKT S473/T308 | Total Akt | PTEN | cIAP1 | cIAP2 | XIAP | Survivin |
|---|---|---|---|---|---|---|---|---|---|
| **KYAE1** | 3.680 | 12.875 | 5.909 | 1.242 | 1.08 | 0.84 | 7.45 | 2.48 | 1.00 |
| **OE19** | 2.320 | 11.000 | 4.424 | 1.159 | 1.12 | 1.00 | 0.00 | 2.25 | 0.77 |
| **OACM5C1** | 2.320 | 1.875 | 2.242 | 1.432 | 1.67 | 1.54 | 0.00 | 1.33 | 6.51 |
| **ESO26** | 1.040 | 7.375 | 2.576 | 1.000 | 0.03 | 1.06 | 1.00 | 1.00 | 6.26 |
| **SKGT4** | 1.000 | 1.000 | 1.000 | 0.917 | 0.30 | 1.31 | 0.00 | 0.21 | 0.64 |
| **OE33** | 0.560 | 0.625 | 0.545 | 0.955 | 1.00 | 2.50 | 10.16 | 0.78 | 0.62 |
| **ESO51** | 0.120 | 0.750 | 0.273 | 0.962 | 1.10 | 0.71 | 0.00 | 2.50 | 3.17 |
| **OACP4C** | 0.0004 | 0.013 | 0.003 | 0.917 | 0.81 | 0.75 | 9.65 | 0.40 | 1.00 |
| **FLO-1** | 0.020 | 0.375 | 0.091 | 1.030 | 0.59 | 0.71 | 13.79 | 0.41 | 0.54 |
| **Pearson Correlation (r) with ALM301 ~IC50 (72h)** | **-0.25** | **-0.11** | **-0.18** | **-0.2** | **0.2** | **-0.31** | **-0.44** | **0.58** | **0.08** |

**Supplementary Table S4**. OAC and normal oesophageal organoid drug sensitivity together with RNA Seq data for EAC organoids. *CAM408 and CAM479 were resistant to ALM301 doses used in the dose response curve and therefore $IC_{50}$ are predicted values. Shading represents the organoids in which ALM301 and BV6 did not synergise.

| | Expression Relative to Vinculin | | | Drug Sensitivity | | |
|---|---|---|---|---|---|---|
| | **BIRC2** | **BIRC3** | **BIRC5** | **ALM301 $IC_{50}$ (μM)** | **BV6 $IC_{10}$ (μM)** | **BV6 $IC_{30}$ (μM)** |
| **CAM277** | 0.57 | 0.20 | 0.32 | 8.0 | 0.01 | 0.06 |
| **CAM401** | 0.09 | 0.08 | 0.10 | 4.6 | 0.04 | 0.36 |
| **CAM408** | 0.07 | 0.10 | 0.04 | 40.0* | 1.52 | 4.35 |
| **CAM486** | 0.50 | 0.28 | 0.67 | 12.6 | 1.25 | 3.35 |
| **CAM479** | 0.30 | 0.53 | 0.10 | 540.0* | 0.06 | 0.19 |
| **NG008** | | | | 4 | 2.1 | 4.8 |
