# Supplementary material for: Inhibition of AKT enhances chemotherapy efficacy and synergistically interacts with targeting of the Inhibitor of apoptosis proteins in oesophageal adenocarcinoma
Source: Sci Rep. 2024 Dec 30;14:32121. doi: 10.1038/s41598-024-83912-4 (PMC11686190; doi:10.1038/s41598-024-83912-4)
Supplement: Supplementary file 3 — Supplementary Information 3. [file 41598_2024_83912_MOESM3_ESM.pptx]

## Slide 1
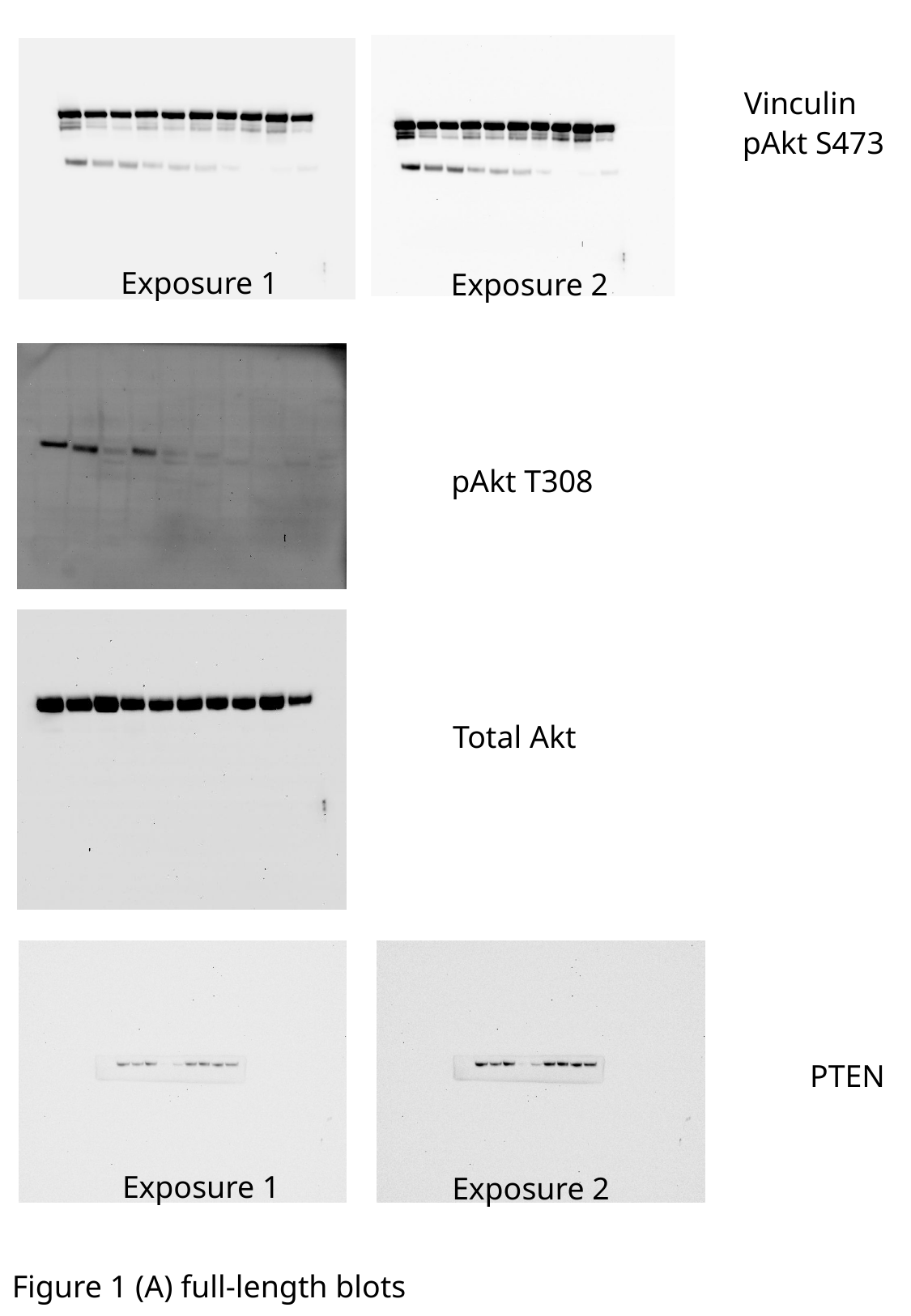

Vinculin
pAkt S473
Exposure 1
Exposure 2
pAkt T308
Total Akt
PTEN
Exposure 1
Exposure 2
Figure 1 (A) full-length blots

## Slide 2
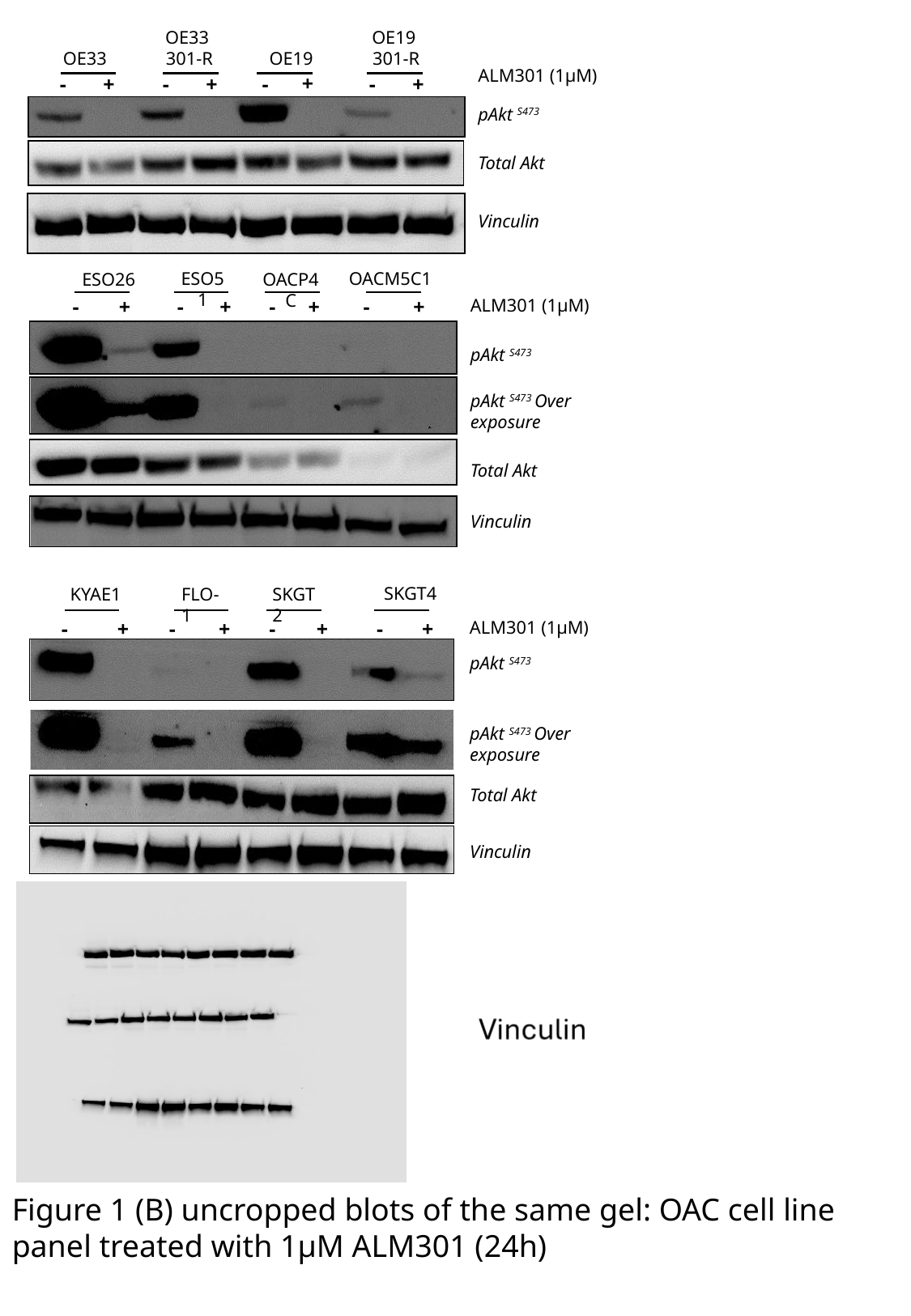

OE33
301-R
OE19
301-R
OE33
OE19
ALM301 (1µM)
+
-
+
-
+
-
-
+
pAkt S473
Total Akt
Vinculin
ESO51
OACM5C1
ESO26
OACP4C
-
+
-
+
-
+
-
+
ALM301 (1µM)
pAkt S473
pAkt S473 Over exposure
Total Akt
Vinculin
SKGT4
KYAE1
FLO-1
SKGT2
-
+
-
+
-
+
-
+
ALM301 (1µM)
pAkt S473
pAkt S473 Over exposure
Total Akt
Vinculin
Figure 1 (B) uncropped blots of the same gel: OAC cell line panel treated with 1µM ALM301 (24h)

## Slide 3
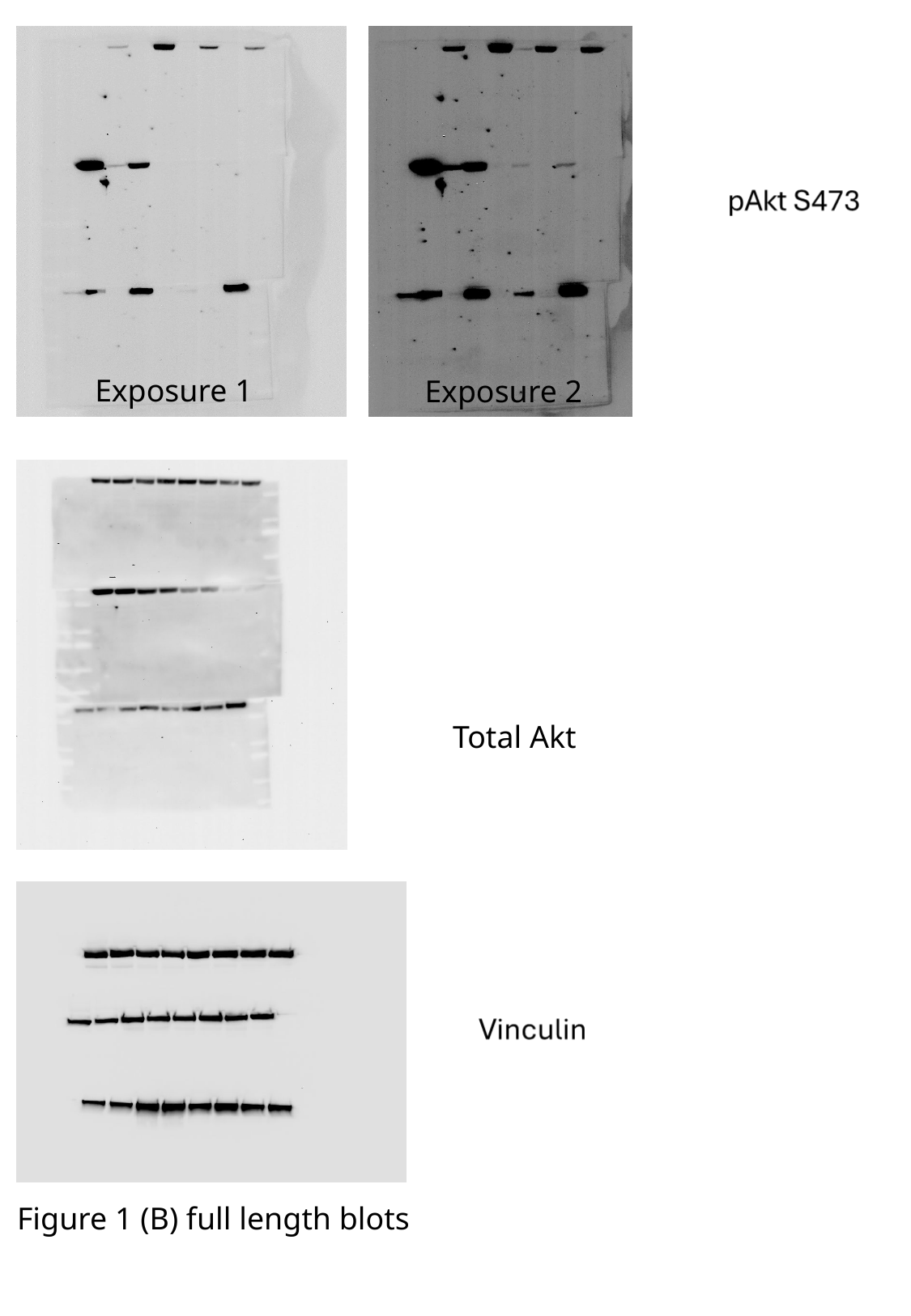

Exposure 1
Exposure 2
Total Akt
Figure 1 (B) full length blots

## Slide 4
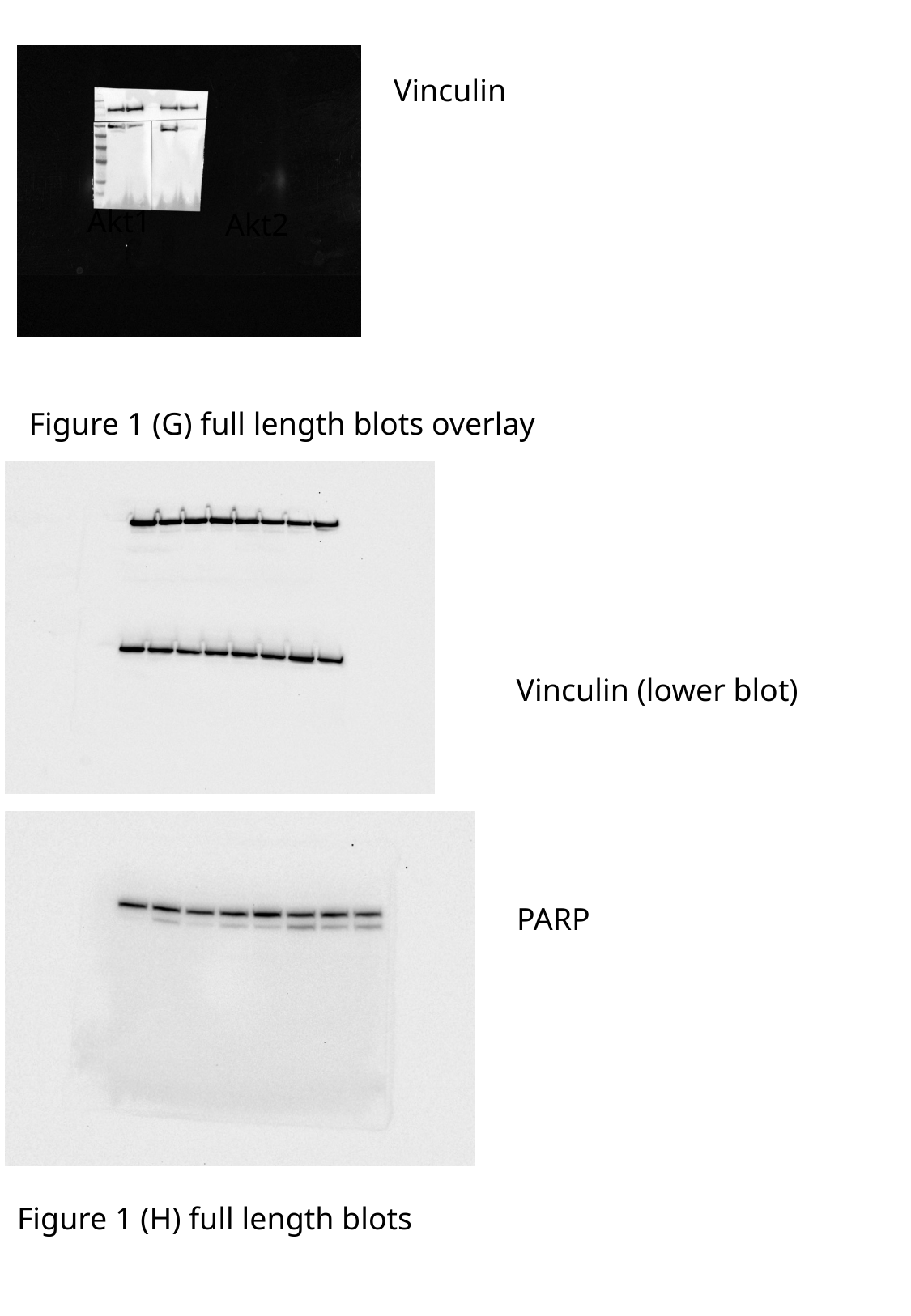

Vinculin
Akt1
Akt2
Figure 1 (G) full length blots overlay
Vinculin (lower blot)
PARP
Figure 1 (H) full length blots

## Slide 5
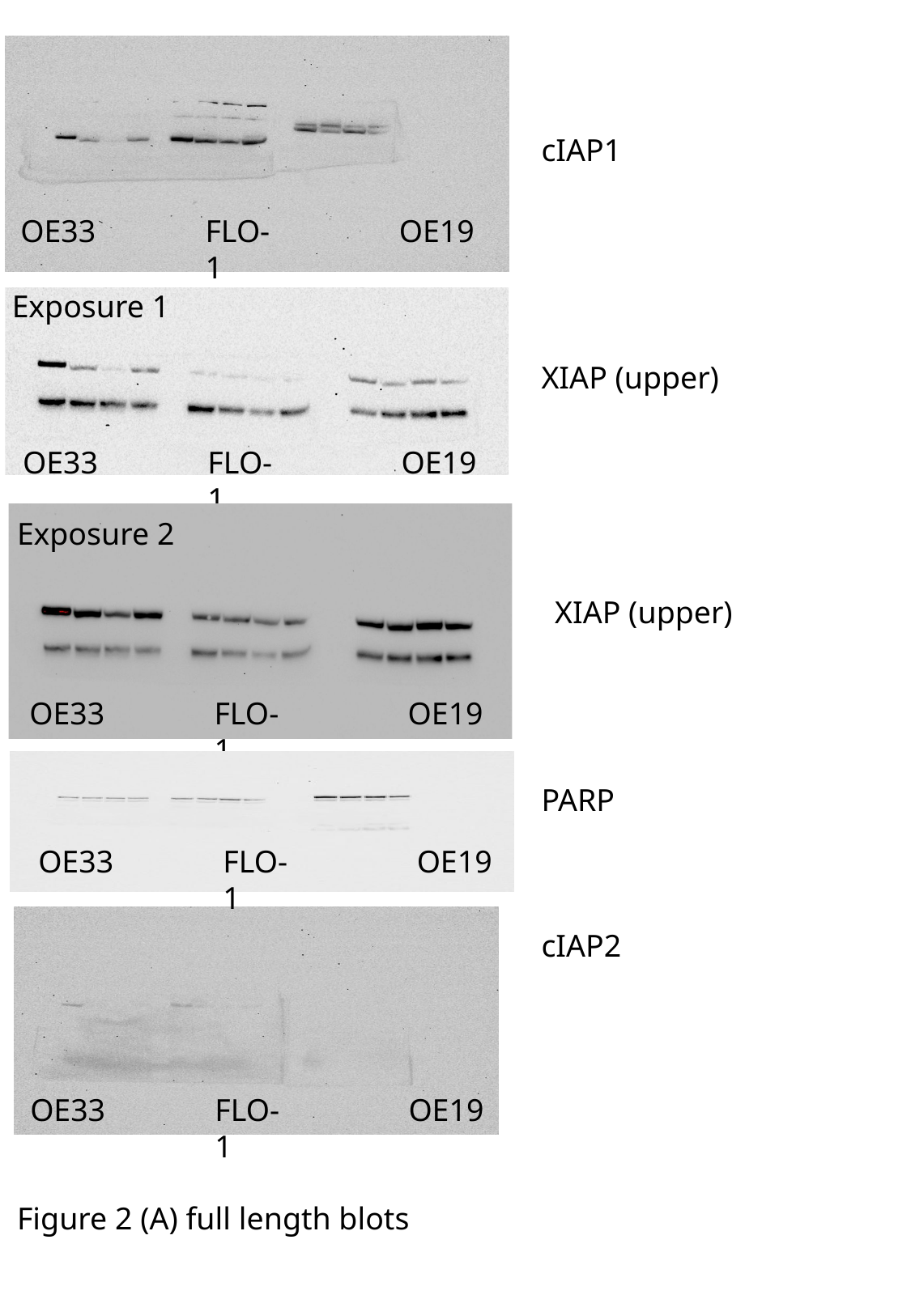

cIAP1
OE33
FLO-1
OE19
Exposure 1
XIAP (upper)
OE33
FLO-1
OE19
Exposure 2
XIAP (upper)
OE33
FLO-1
OE19
PARP
OE33
FLO-1
OE19
cIAP2
OE33
FLO-1
OE19
Figure 2 (A) full length blots

## Slide 6
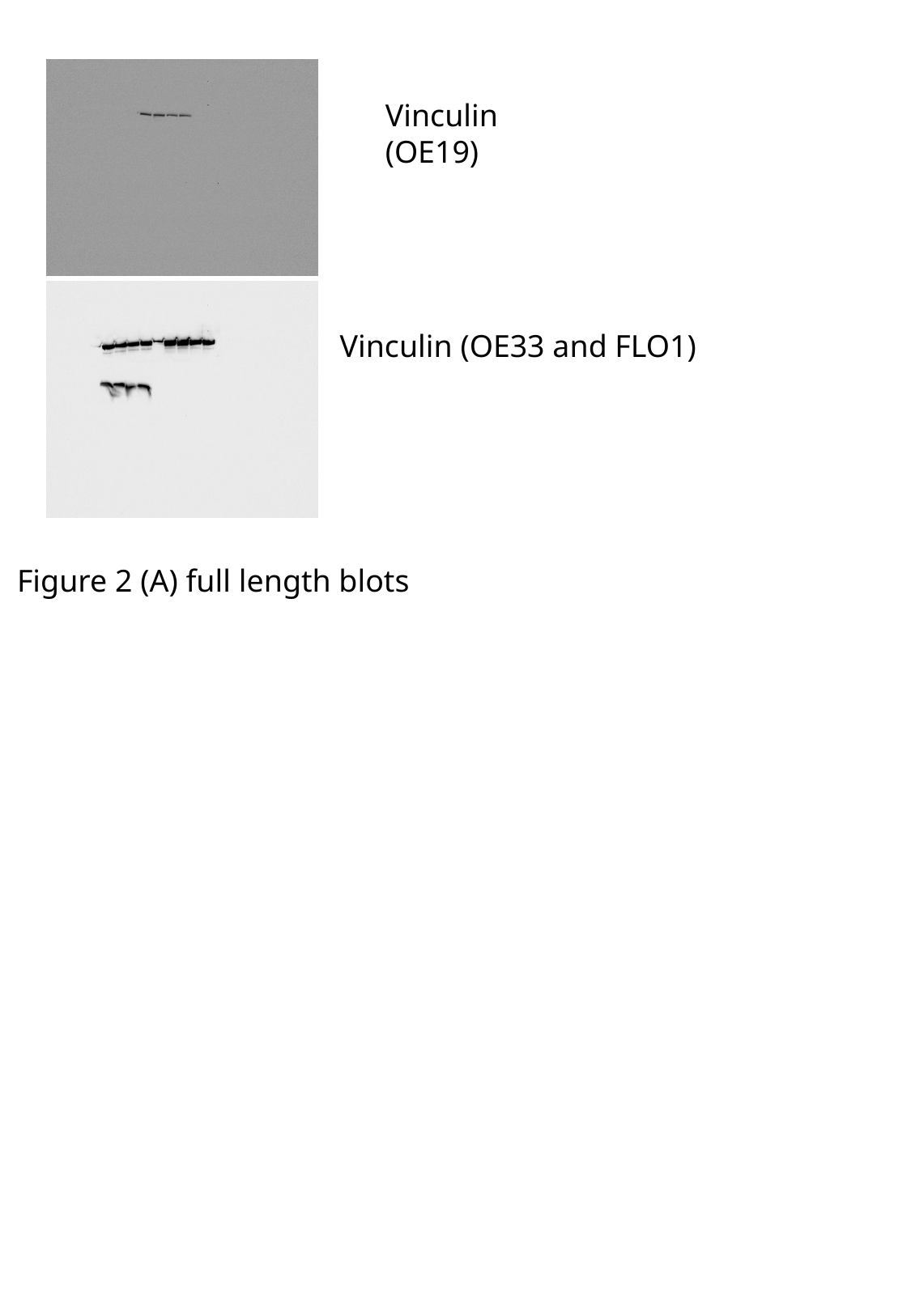

Vinculin (OE19)
Vinculin (OE33 and FLO1)
Figure 2 (A) full length blots

## Slide 7
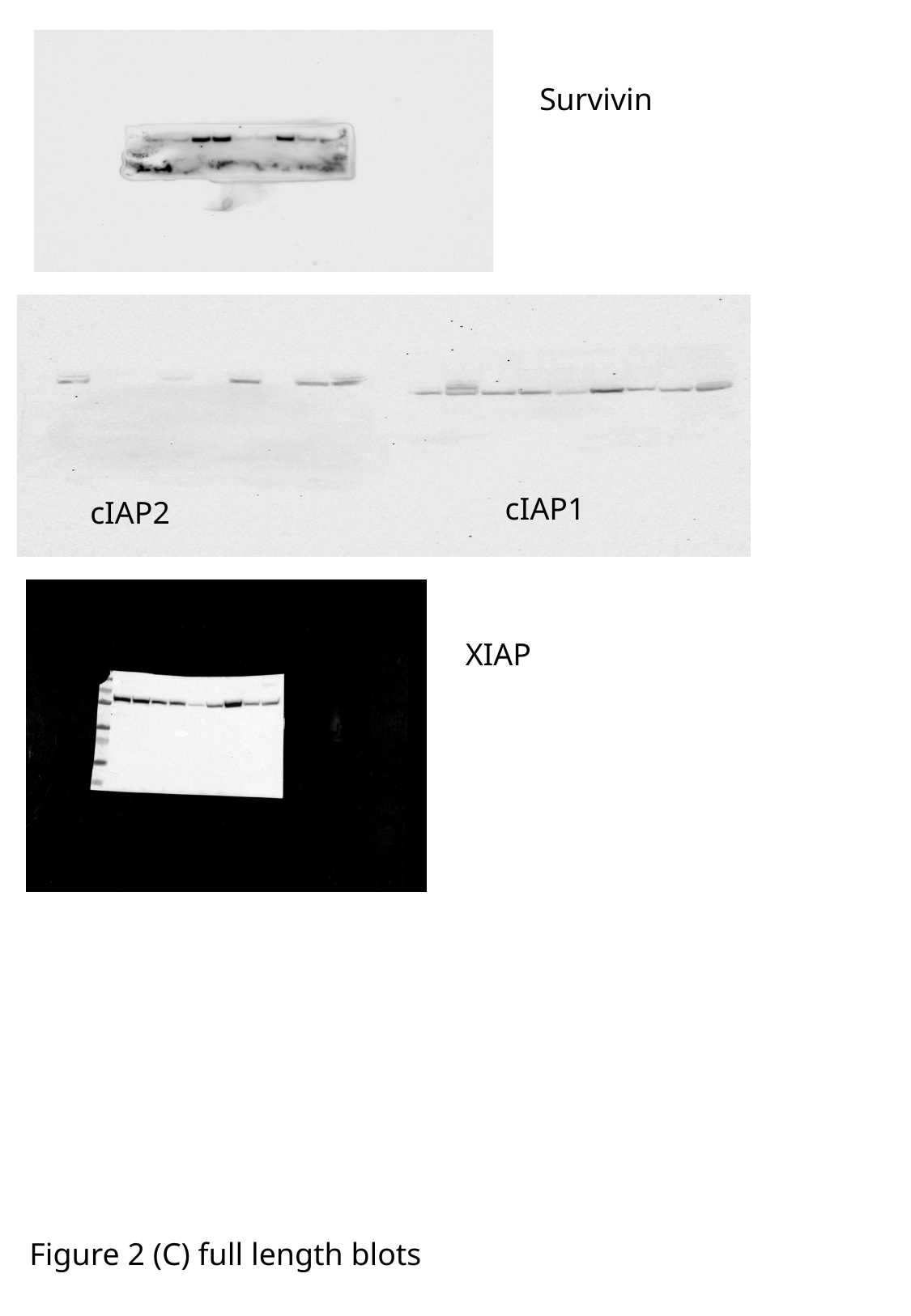

Survivin
cIAP1
cIAP2
XIAP
Figure 2 (C) full length blots

## Slide 8
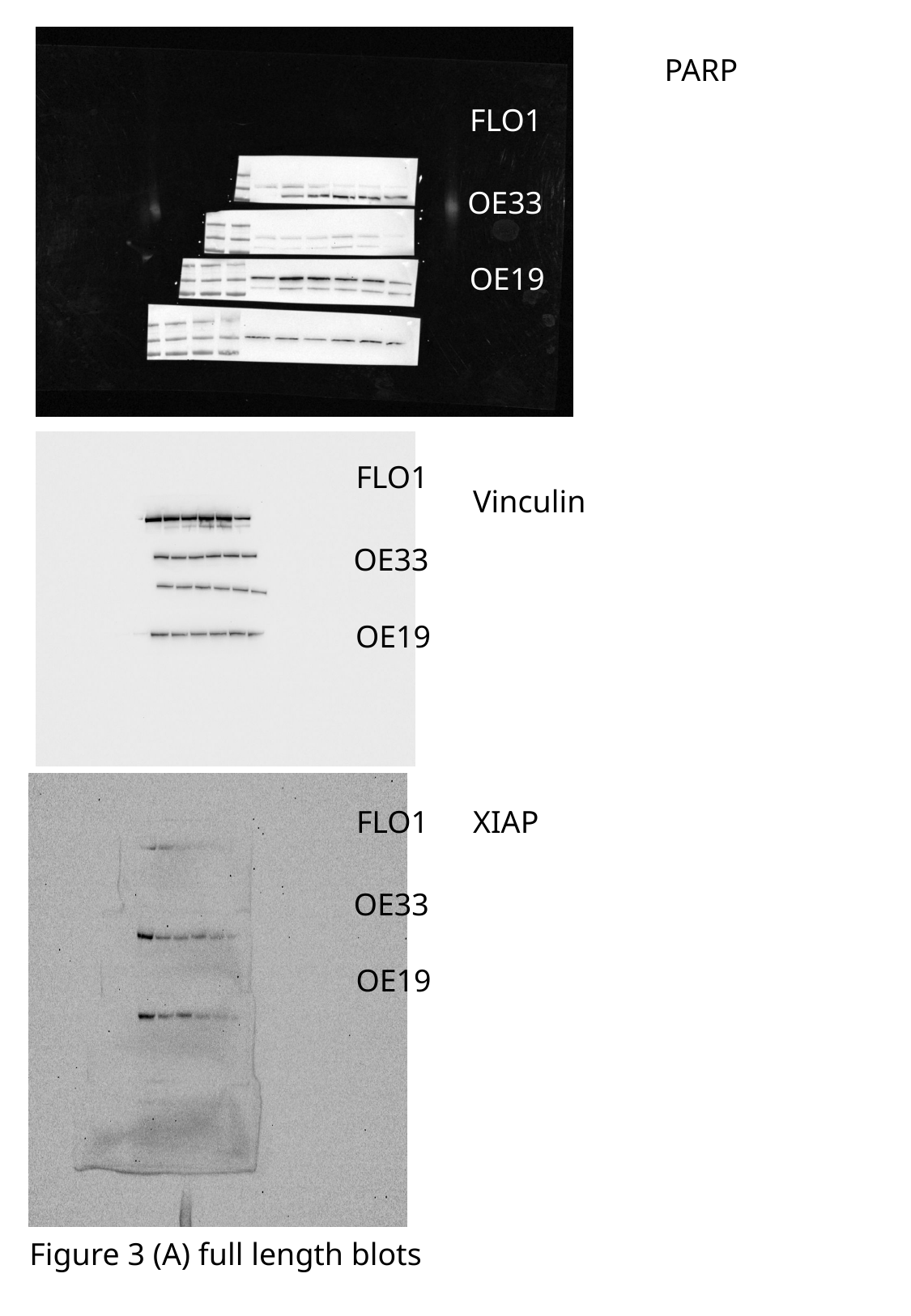

PARP
FLO1
OE33
OE19
FLO1
Vinculin
OE33
OE19
FLO1
XIAP
OE33
OE19
Figure 3 (A) full length blots

## Slide 9
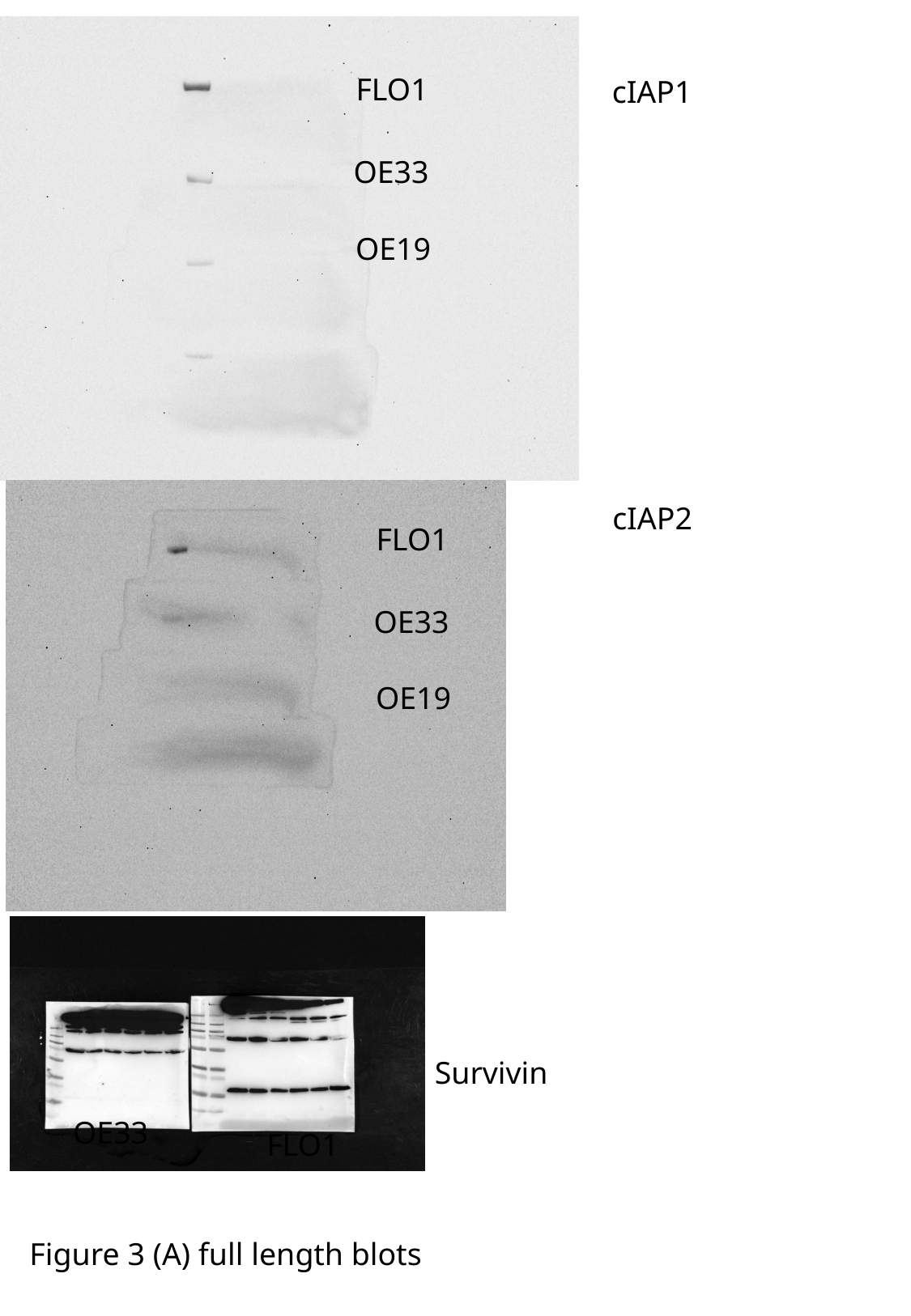

FLO1
cIAP1
OE33
OE19
cIAP2
FLO1
OE33
OE19
Survivin
OE33
FLO1
Figure 3 (A) full length blots
